# An overview of the synthetic routes to the best selling drugs containing 6-membered heterocycles

**DOI:** 10.3762/bjoc.9.265

**Published:** 2013-10-30

**Authors:** Marcus Baumann, Ian R Baxendale

**Affiliations:** 1 Department of Chemistry, University of Durham, South Road, Durham, DH1 3LE, UK

**Keywords:** heterocycles, medicinal chemistry, pharmaceuticals, six-membered rings, synthesis

## Abstract

This review which is the second in this series summarises the most common synthetic routes as applied to the preparation of many modern pharmaceutical compounds categorised as containing a six-membered heterocyclic ring. The reported examples are based on the top retailing drug molecules combining synthetic information from both scientific journals and the wider patent literature. It is hoped that this compilation, in combination with the previously published review on five-membered rings, will form a comprehensive foundation and reference source for individuals interested in medicinal, synthetic and preparative chemistry.

## Introduction

The commonality of six-membered heterocyclic rings in pharmaceutical actives inevitably generates a substantial body of reference material. Consequently this review has been broadly partitioned into six consecutive sections categorised by analogous groupings of heterocyclic rings.

The subsections are:

1. Pyridines and quinolines
2. Dihydropyridines and piperidines
3. Pyrimidines and quinazolines
4. Pyrazines and piperazines
5. Pyridazines and perhydropyridazines
6. Triazines and polyazacyclic systems

Synthetic chemistry can rightfully be considered a prerequisite of our modern society [1]. This discipline supplies many valuable resources to our world enabling us to produce the quantities of fertilizer needed to feed a growing world’s population and produce the numerous customised materials without which society could not progress. Importantly, synthetic chemistry has had a huge impact on public health where treatments for almost any disease can be developed resulting in a steady increase in life expectancy [2]. All these advances have been enabled by the curiosity of generations of scientists constantly searching for new solutions to the assembly of functional molecules. Importantly this has required the development of several new methods for selectively forming new chemical bonds allowing the generation of more complex drug candidates [3–4]. However, one downside of drug research lies in the immense cost of the development and regulatory processing of a new drug with only 15–20 years of commercial protection being granted to recoup this initial outlay [5]. As a result pharmaceutical companies are constantly seeking ways to accelerate this development process by adopting new synthetic methodologies and enabling technologies in order to profitably generate new medications for both old and new targets [6]. By reviewing the synthetic routes used to construct modern pharmaceutical structures a general overview of the most valuable synthetic techniques and best working practices as used by the pharmaceutical industry can be constructed.

Previously, we reviewed the most common synthetic routes to the top-marketed drugs containing heterocyclic five-membered rings, which allowed us to analyse how medicinal chemists have addressed challenges over the past 30 years [7]. This work not only represents an overview of the various chemical transformations employed, but also allowed us to judge whether and to what extent new ideas and concepts have been harvested to enhance success rates and accelerate development times of drugs. In this new review article we wish to present a complementary compilation of the synthesis routes of current drugs containing six-membered heterocyclic rings. We believe that the combination of aromatic as well as non-aromatic six-membered structures will give a comprehensive overview that will be of value to any student, academic or future medicinal chemist interested in applied chemical synthesis.

It should be noted that the heterocycles discussed are named according to IUPAC recommendations wherever possible; however, in some instances different names are commonly used by the synthetic community (i.e. pyridone and pyridinone). Furthermore, in most cases throughout this review only the syntheses of the free-based form of the parent drugs is discussed although these are commonly formulated in their prescription form as salts.

**Table 1:** Chemical structures of the heterocyclic compounds discussed in this review.

Name	Label	Structure	Heterocycle	Scheme

Nicotinic acid	**1.12**	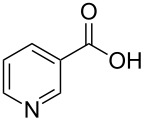	pyridine	2,3
Niacin	**1.18**	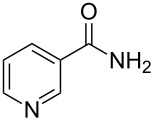	pyridine	3
Clarinex	**1.22**	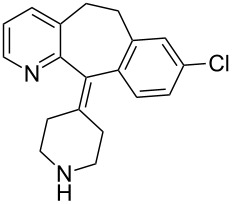	pyridine	5
Rabeprazole	**1.33**	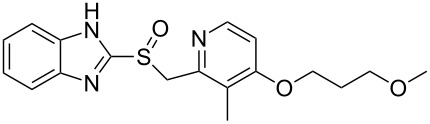	pyridine	6,7
Pantoprazole	**1.34**	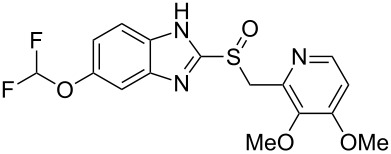	pyridine	6,7
Lansoprazole	**1.35**	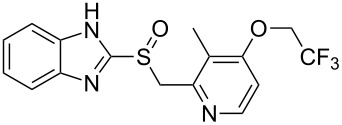	pyridine	6,7
Rosiglitazone	**1.40**	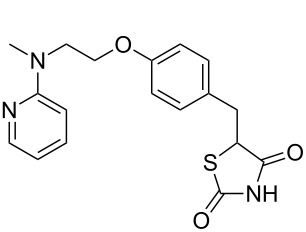	pyridine	8,11
Pioglitazone	**1.41**	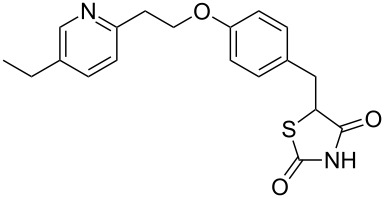	pyridine	12–16
Etoricoxib	**1.90**	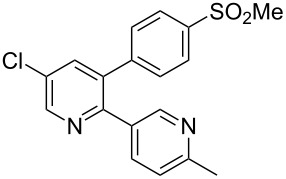	pyridine	17,18
Moxifloxacin	**1.102**	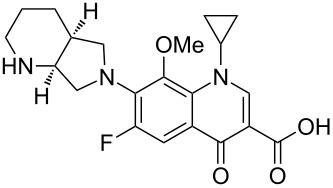	quinolone	19,20
Levofloxacin	**1.103**	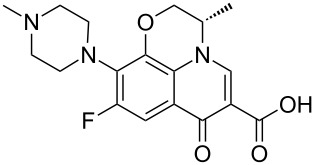	quinolone	21,22
Nifedipine	**2.1**	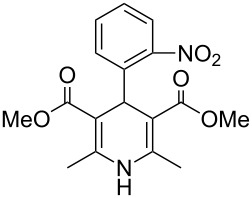	dihydropyridine	23
Amlodipine	**2.2**	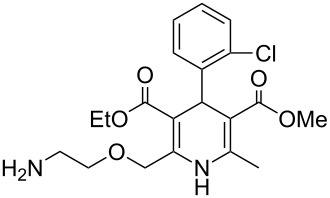	dihydropyridine	24,25
Clevidipine	**2.3**	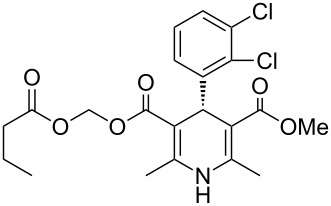	dihydropyridine	26
Tiagabine	**2.36**	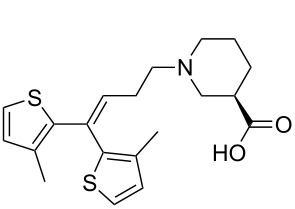	piperidine	27
Solifenacin	**2.57**	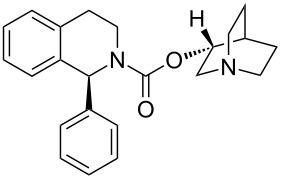	piperidine	28
Carmegliptine	**2.70**	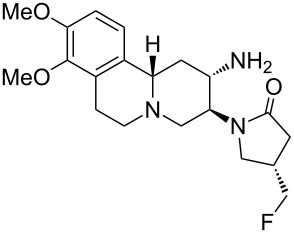	piperidine	30
Lamivudine	**3.1**	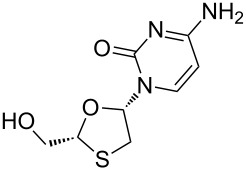	pyrimidine	33
Raltegravir	**3.18**	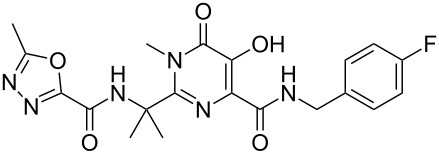	pyrimidine	34
Imatinib	**3.36**	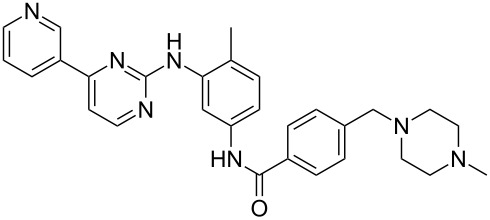	pyrimidine	37,38
Erlotinib	**3.37**	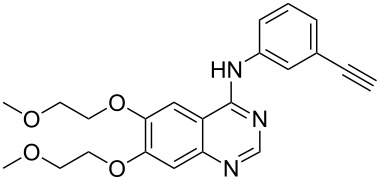	pyrimidine	39,40
Lapatinib	**3.38**	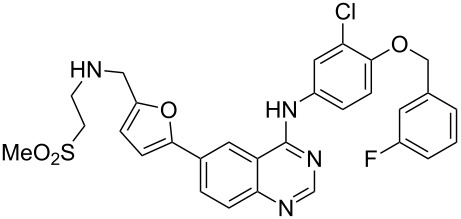	pyrimidine	41
Rosuvastatin	**3.80**	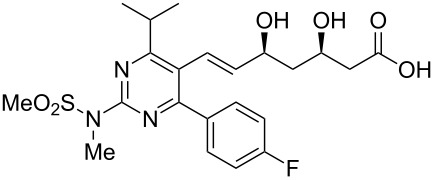	pyrimidine	42,43
Varenicline	**4.1**	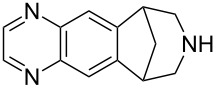	pyrazine	44
Eszopiclone	**4.26**	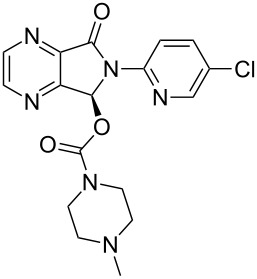	pyrazine	45
Brimonidine	**4.22**	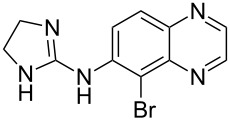	pyrazine	45
Bortezomib	**4.27**	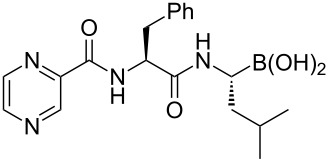	pyrazine	46
Aplaviroc	**4.37**	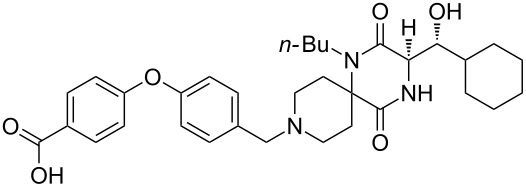	piperazine	47
Azelastine	**5.1**	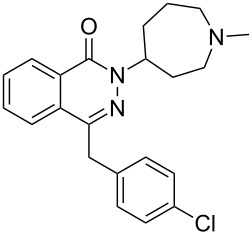	pyridazine	48
Cilazapril	**5.12**	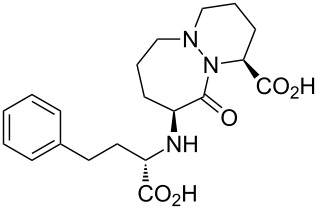	perhydro-pyridazine	49
Lamotrigine	**6.1**	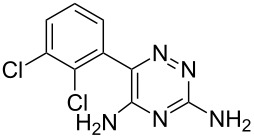	1,2,4-triazine	50,51
Imiquimod	**6.11**	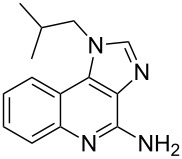	imidazoquinoline	52–54
Abacavir	**6.37**	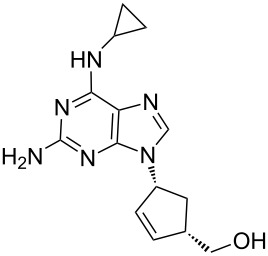	purine	55
Ocinaplon	**6.48**	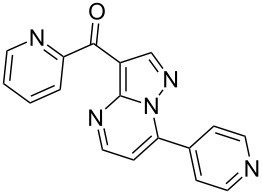	pyrazolo-pyrimidine	56
Zaleplon	**6.46**	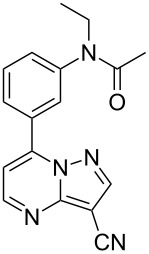	pyrazolo-pyrimidine	57
Indiplon	**6.54**	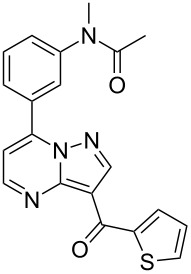	pyrazolo-pyrimidine	57
Sildenafil	**6.61**	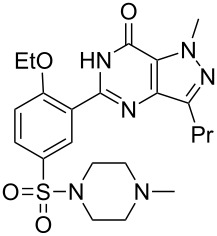	pyrazolo-pyrimidone	58–61
Vardenafil	**6.87**	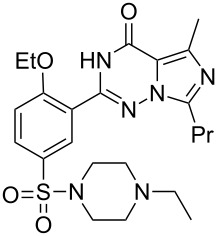	imidazotriazinone	62–65
Temozolomide	**6.105**	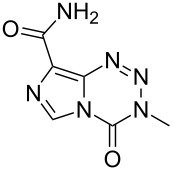	imidazo-tetrazinone	66–68

## Review

### 1. Pyridines and quinolines

The pyridine ring can be considered as one of the simplest yet most important heteroaromatic structures. Naturally occurring in many important compounds such as the vitamins niacin and pyridoxine, the ubiquitous redox system NADP/NADPH and a number of alkaloids including nicotine, pyridine, is thus important for a wide range of biological activities [8]. Consequently, the pyridine ring is utilised in many pharmaceutical actives and possibly even more commonly found in agrochemical products. This can be rationalised by the fact that simple pyridines readily undergo metabolism via oxidation or methylation pathways forming the corresponding pyridinium ions [9–10]. Although many of these metabolites are potentially highly toxic to humans they are conveniently and quickly excreted from the body via the kidneys. With a good understanding of the pharmacokinetics and distribution profile pyridines can therefore be tolerated in the context of pharmaceuticals. Often pyridines can be made more resilient to metabolic changes by increasing their functionalisation or electronically biasing them against direct oxidation. However, the modern trend in structural optimisation of pyridine containing lead compounds is to commonly replace the ring with a bioisostere such as a methylisoxazole, isothiazole, oxadiazole [11–12] or various diazines [13–14]. This strategy is based mainly upon a desire to avoid potential late stage toxicology issues which as indicated have arisen in the pyridine structural class.

**Scheme 1:**
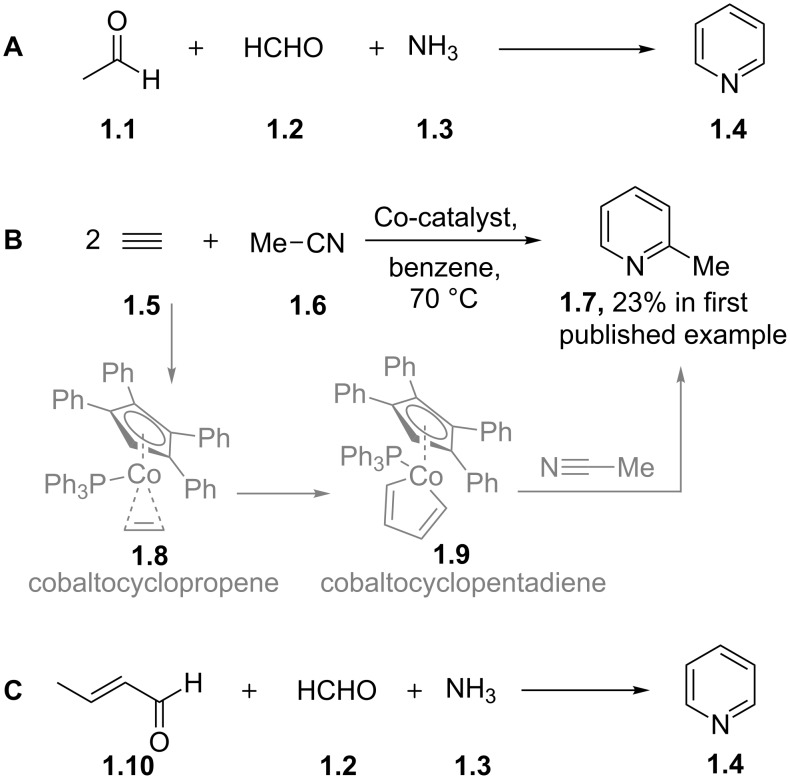
Scaled industrial processes for the synthesis of simple pyridines.

Pyridine itself is produced industrially by either the traditional Chichibabin pyridine synthesis (Scheme 1, A), the Bönnemann reaction, a cobalt-catalysed cyclotrimerisation of alkynes and nitriles (Scheme 1, B) or the aerobic gas-phase condensation of croton aldehyde, formaldehyde and ammonia (Scheme 1, C). Likewise numerous methods of synthesising substituted pyridines have been reported [15]. Much of this development was stimulated by the discovery of pharmaceutically active pyridine containing biogenic structures but mainly in an industrial context by the drive for new polymeric materials such as the vinylpyridine based latex in the first half of the 20th century.

An excellent summative review concerning the general synthesis of pyridines has recently been published [16]. From this source and several others it is evident that the most versatile preparative method for substituted pyridines is the direct condensation of ammonia (or hydroxylamine) with a corresponding 1,5-diketone. Alternatively, ammonia (or an ammonium salt), an aldehyde and two equivalents of a 1,3-dicarbonyl compound can react via a classical Hantzsch dihydropyridine synthesis. Similarly unsymmetrical pyridines can be furnished by employing an amino-enone or aminonitrile compound with a 1,3-dicarbonyl moiety. In both cases a subsequent oxidation step is usually required to convert the initially formed dihydropyridine intermediate into the desired aromatic pyridine. Some additional but much less common methods utilising cycloadditions of electron-rich oxazoles and acrylates [17–18] tri- and tetrazines with alkenes/alkynes [19] or pyrones with nitriles [20] have been used successfully to access specifically functionalised pyridines which are difficult to prepare by the more direct condensation routes.

A major synthetic obstacle to accessing diversely substituted pyridines is that each preparation must start from a set of chemically distinct building blocks that often require significant optimisation to determine the most appropriate reaction conditions for their condensation. Despite this, combinatorial chemistry techniques have been used to generate large compound libraries in the hope that high-throughput screenings would identify new structures worthy of further development [21]. Unfortunately, these efforts have not resulted in a net increase in the number of new pyridine containing drug candidates but have yielded structurally useful data regarding many physical properties such as p*K*_b_, and oxidation stability.

When examining the synthetic origins of pyridine substructures represented in the top-market API’s (active pharmaceutical ingredients) a distinct division can be easily recognised. Principally this pertains to those pyridine units installed from a generic commercially available building block and pyridines specifically prepared via de novo synthesis with the intent of tailoring the physical or metabolic stability of the compound. Often in the latter case the pyridine ring is an integral part of the pharmacophore rather than simply imparting a modicum of increased solubility or tuning a parameter such as Log*P* [22].

An important example of de novo synthesis of the pyridine core can be found in the syntheses of nicotinic acid (**1.12**, vitamin B3/niacin) and nicotinamide (**1.18**, constituent of coenzyme NAD{P}). Although these entities are biogenic, the human body is not capable of producing them, thus industrial processes have had to be developed, Lonza and Reilly are historically the key players in this area [23].

**Scheme 2:**
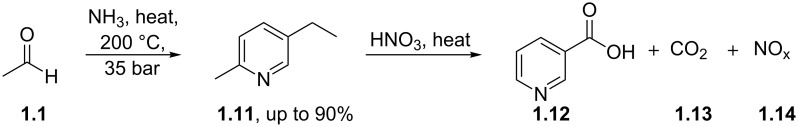
Synthesis of nicotinic acid from 2-methyl-5-ethylpyridine (**1.11**).

The molecule 2-methyl-5-ethylpyridine (**1.11**) can be prepared directly via a condensation between acetaldehyde and ammonia, nitric acid mediated oxidation then converts this to nicotinic acid (**1.12**) [24]. Although the initial pyridine formation is high yielding, this process is not without environmental issues due to the large excess of nitric acid and high temperatures combined with the liberation of large amounts of gases such as nitric oxides and carbon dioxide which must be scrubbed from the system (Scheme 2). Interestingly, despite these considerations this method of synthesis is currently still run by the speciality chemicals company Lonza but now as a continuous flow process at their main plant in Visp, Switzerland.

**Scheme 3:**
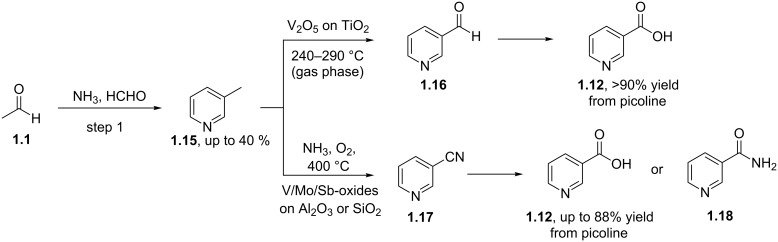
Synthesis of 3-picoline and nicotinic acid.

An alternative process is based on the availability of 3-picoline (**1.15**) which is generated as a major side product in the synthesis of pyridine prepared from formaldehyde, acetaldehyde and ammonia in a gas phase reaction (Scheme 3) [25]. The 3-picoline can be readily oxidised via another gas-phase protocol using a fixed-bed reactor charged with vanadium pentoxide on high surface titanium dioxide (5–50 wt % vanadium). A modification of the sequence utilises a dehydrative amminolysis (ammoxidation) to furnish the corresponding 3-cyanopyridine, which can then be subsequently hydrolysed to nicotinic acid. The catalyst systems most commonly used in this high temperature ammoxidation are based on vanadium, molybdenum or antimony oxides supported on silica or alumina.

**Scheme 4:**
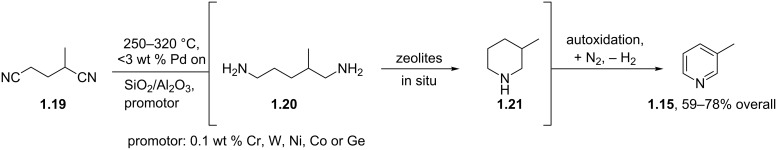
Synthesis of 3-picoline from 2-methylglutarodinitrile **1.19**.

Since the initial Chichibabin type sequence (step 1; Scheme 3) leading to 3-picoline is not an entirely selective process, alternative strategies starting from other bulk materials have been devised. For example, taking a stream of 2-methylpentane-1,5-diamine (**1.20**), a derivative of 2-methylglutaronitrile **1.19** (a side product of adiponitrile production) can be cyclised to yield 3-methylpiperidine (**1.21**) using zeolites and easily aromatised by catalytic dehydrogenation to 3-picoline in 78% overall yield (Scheme 4) [26]. The overall processing sequence is highly energy-efficient (coupling of the endothermic cyclisation with the exothermic dehydration gives a reasonable energy balance). In addition the ammonia liberated during the cyclisation step is later consumed in the ammoxidation of the 3-picoline to the corresponding 3-cyanopyridine (Scheme 3).

**Scheme 5:**
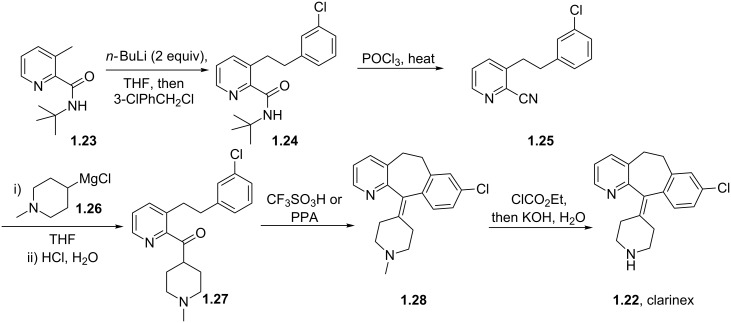
Picoline-based synthesis of clarinex (no yields reported).

The value of substituted 3-picoline precursors is illustrated in the synthesis of clarinex (**1.22**, Desloratadine, Scheme 5), a dual antagonist of platelet activating factor (PAF) and of histamine used in the treatment of allergies. This compound consists of a highly functional tricyclic core with an unsaturated linkage to a pendant piperidine ring. The picoline derivative **1.23** is first treated with two equivalents of *n*-butyllithium (*n*-BuLi) followed by alkylation with benzyl chloride to give the chain elongated adduct [27]. The *tert*-butylamide **1.24** is then dehydrated with phosphorous oxychloride at elevated temperatures to yield the nitrile derivative **1.25**. Introduction of the piperidine ring is achieved by utilisation of the appropriately substituted Grignard reagent **1.26**. A Friedel–Crafts type acylation promoted by either triflic acid or polyphosphoric acid (PPA) furnishes the tricyclic structure **1.28** which upon *N*-demethylation affords clarinex (**1.22**).

**Scheme 6:**
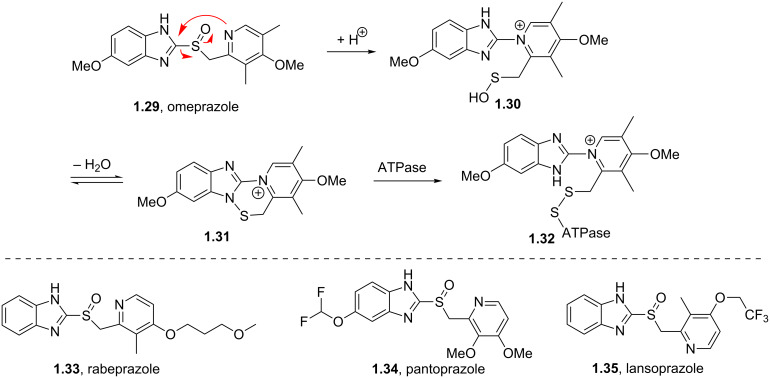
Mode of action of proton-pump inhibitors and structures of the API’s.

One of the top-selling classes of pharmaceuticals containing the pyridine ring are the proton pump inhibitors; numerous examples such as omeprazole (**1.29**, Losec), rabeprazole (**1.33**, Aciphex), pantoprazole (**1.34**, Protonix) and lansoprazole (**1.35**, Prevacid) populate this area [28]. All these API’s contain the characteristic benzimidazole unit bearing a sulfoxide substituent at the 2-position. Interestingly all these compounds are actually pro-drugs hence the common structural features can be rationalised as the molecules undergo an acid-catalysed Smiles-rearrangement prior to bioconjugation to ATPases (Scheme 6).

**Scheme 7:**
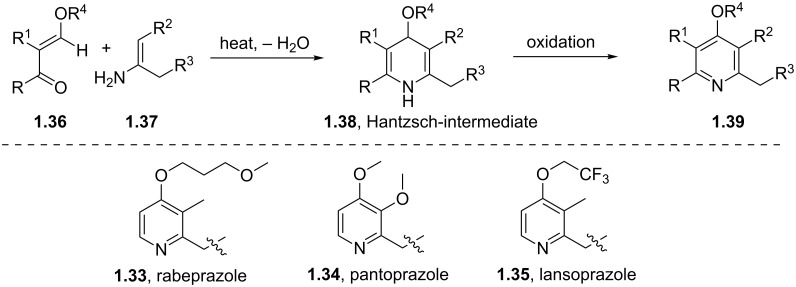
Hantzsch-like route towards the pyridine rings in common proton pump inhibitors.

Most of the synthetic routes towards the embedded pyridine heterocycle in these molecules are not reported thoroughly as the pyridine subunit is typically introduced as a commercially available building block. Nevertheless we can assume that most strategies likely employ a modification of the traditional Hantzsch synthesis as outlined in Scheme 7 [29]. Here 1,3-disubstituted ketone derivatives are treated with α,β-unsaturated carbonyl compounds in the presence of ammonia to initially form dihydropyridines **1.38**, which can ultimately be converted into the corresponding aromatic pyridine upon oxidation with a variety of oxidants such as MnO_2_, HNO_2_, HNO_3_ or cerium ammonium nitrate (CAN). The mechanism of the classical Hantzsch reaction has been studied extensively by numerous groups and culminated in an article by Katrizky and coworkers describing their findings based on ^13^C and ^15^N NMR spectroscopic evidence. From this it can be concluded that an enamine species **1.37** is initially formed and subsequently undergoes cyclocondensation with the Michael acceptor **1.36** as the rate determining step of this sequence [30].

**Figure 1:**
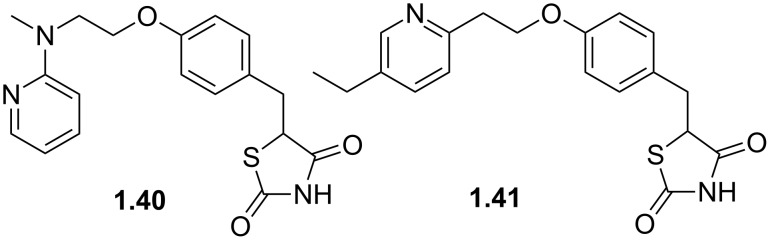
Structures of rosiglitazone (**1.40**) and pioglitazone (**1.41**).

Other important species containing a pyridine moiety are rosiglitazone (**1.40**, Avandia) and pioglitazone (**1.41**, Actos), which are members of the so called thiazolidinedione class of type-2 diabetes drugs (Figure 1). These pharmaceutical agents act as binders to the peroxisome proliferator-activated receptors that upon activation migrate to the DNA to regulate the transcription of specific genes which control the metabolism of carbohydrates and fatty acids. The structures of pioglitazone and rosiglitazone show common structural features bearing a distal pyridine ring linked to the thiazolidinedione pharmacophore.

**Scheme 8:**
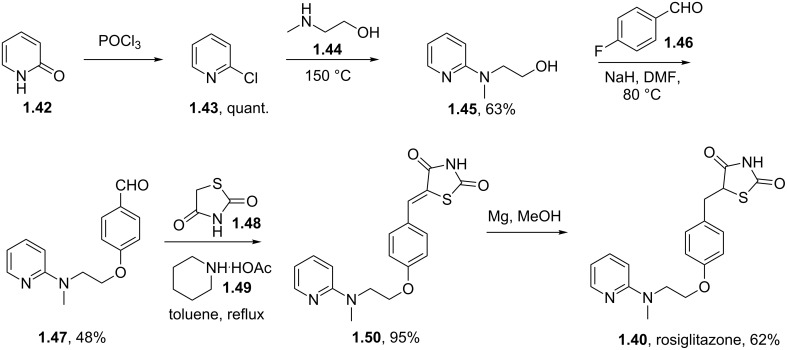
Synthesis of rosiglitazone.

In rosiglitazone the pyridine unit is introduced via an S_N_Ar reaction between *N*-methylethanolamine (**1.44**) and 2-chloropyridine (**1.43**) which in turn is readily prepared by chlorination of 2-pyridone (**1.42**) with phosphorous oxychloride (Scheme 8) [31–32]. The resulting primary alcohol **1.45** is then subjected to a second S_N_Ar reaction with 4-fluorobenzaldehyde [33]. A Knoevenagel condensation of the aldehyde functionality in compound **1.47** with thiazolidinedione **1.48** in the presence of a catalytic amount of piperidinium acetate reportedly leads to the exclusive formation of the desired *Z*-isomer product. Interestingly, the newly installed double bond was efficiently reduced using magnesium in methanol thus circumventing catalyst poisoning issues pertaining to the thiazolidinedione moiety as experienced using other reducing systems.

**Scheme 9:**
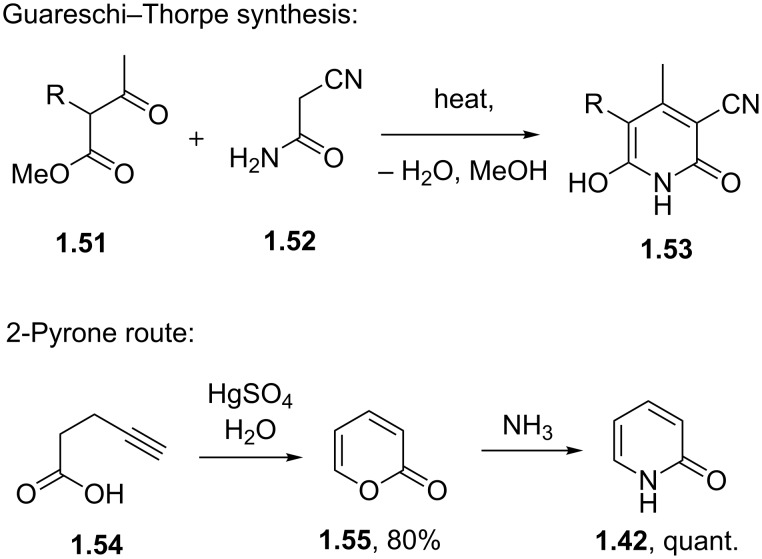
Syntheses of 2-pyridones.

As an aside the synthesis of 2-pyridones (i.e. **1.42**) can be achieved via a number of methods. For example the classical Guareschi–Thorpe condensation in which cyanoacetamide reacts with a 1,3-diketone delivers highly substituted 2-pyridones (Scheme 9) [34–35]. This protocol is closely related to the Hantzsch pyridine synthesis and offers access to a wide range of products with well-defined regioselectivity. The simple undecorated parent 2-pyridone (**1.42**) can be somewhat harder to access but is obtained in a linear sequence via the corresponding 2-pyrone (e.g. Scheme 9), which is converted to the 2-pyridone through an exchange with ammonia or an equivalent nitrogen source (NH_4_X; X = Cl, Br, I, OAc, OH).

**Scheme 10:**
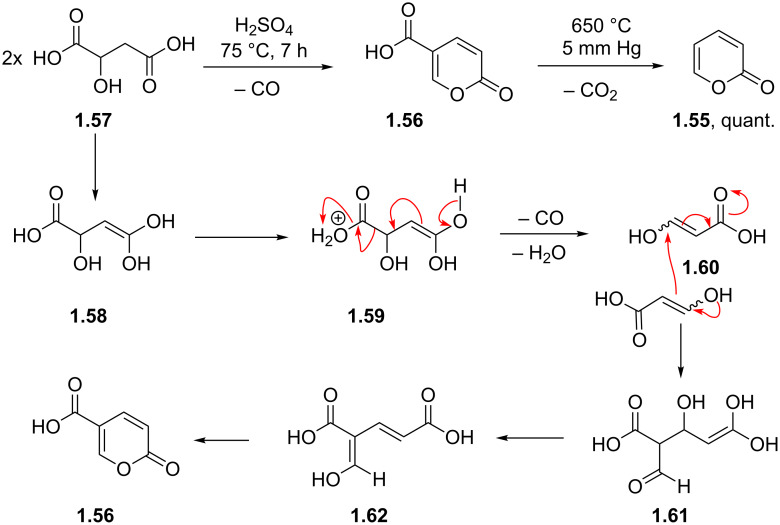
Synthesis and mechanism of 2-pyrone from malic acid.

However, more conveniently the 2-pyrone precursor can be generated via the vacuum pyrolysis of coumalic acid (**1.56**) [36]. Cheap and readily available malic acid (**1.57**) undergoes self-condensation to yield coumalic acid under strongly acidic dehydrating conditions [37–40]. The mechanism is believed to progress by initial dehydration/decarbonylation of malic acid to give an aldehyde acid enol **1.60** which then condenses by Michael addition of the enol to the corresponding enone to give after further dehydration and lactonisation coumalic acid (Scheme 10).

**Scheme 11:**
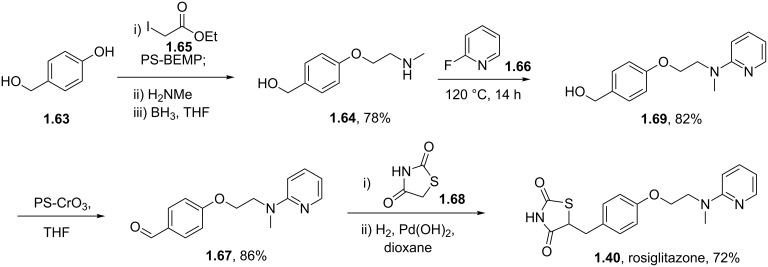
Polymer-assisted synthesis of rosiglitazone.

Returning to the main discussion, a recent synthesis of rosiglitazone [41] exemplifies the advantages of employing resin-bound agents in order to increase yields and purities of intermediates and products in the sequence (Scheme 11). Starting from 4-(hydroxymethyl)phenol (**1.63**) the common *N*-methylamine intermediate **1.64** was obtained in a high yielding three step sequence. First a phenolic alkylation reaction on ethyl 2-iodoacetate (**1.65**) promoted by the strong P1 base polymer-supported BEMP gave the corresponding ether adduct which was then converted to the desired secondary amine **1.64** via direct amidation and borane-mediated reduction. Next an S_N_Ar reaction on 2-fluoropyridine (**1.66**) followed by oxidation of the benzylic alcohol using immobilised chromium(VI) oxide yielded the aldehyde **1.67** required for the previously described Knoevenagel condensation. The five membered heterocycle 2,4-thiazolidinedione (**1.68**) is readily available commercially, but can be easily prepared at scale via a simple cyclocondensation between thiourea and chloroacetic acid [42]. Finally, hydrogenation of the double bond using Pearlmans catalyst furnishes rosiglitazone (**1.40**).

**Scheme 12:**
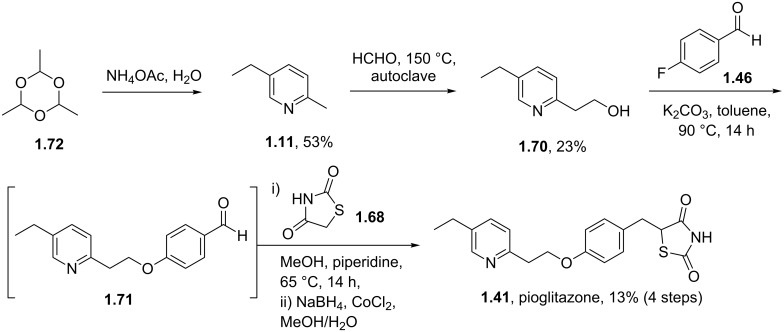
Synthesis of pioglitazone.

In addition to the illustrated syntheses of rosiglitazone several routes towards pioglitazone (**1.41**) and its key intermediates have been reported. Pioglitazone is clearly related to rosiglitazone (**1.40**) with its structure only differing in the substitution pattern of the parent pyridine ring. Its synthesis begins with the hydroxymethylation of 2-methyl-5-ethylpyridine (**1.11**), a commodity chemical obtained from the condensation of acetaldehyde with ammonium acetate (Scheme 12) [43]. At elevated temperatures and pressures 2-methyl-5-ethylpyridine undergoes a condensation reaction with formaldehyde allowing isolation of the chain extended hydroxyethylpyridine **1.70** upon distillation although in poor yield [44]. Following subsequent S_N_Ar reaction aryl ether **1.71** is obtained, which is used as crude material in the subsequent Knoevenagel condensation with thiazolidinedione **1.68**. In order to reduce the intermediate benzylidene double bond in this example sodium borohydride is used in the presence of cobalt chloride efficiently delivering pioglitazone in high purity.

**Scheme 13:**
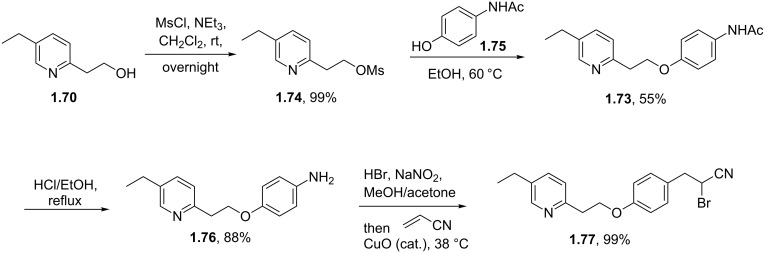
Meerwein arylation reaction towards pioglitazone.

Other syntheses of pioglitazone use related pyridine building blocks and aim to generate late stage intermediates that deliver the target compound upon de novo synthesis of the thiazolidinedione ring system. For instance phenyl ether **1.73** can be obtained via Williamson ether synthesis between mesylate **1.74** and phenol **1.75** (Scheme 13). Removal of the acetyl protecting group under acidic conditions renders aniline **1.76** which is subsequently subjected to a Meerwein arylation reaction which occurs via diazotisation and subsequent treatment with acrylonitrile in the presence of cuprous oxide [45–48].

**Scheme 14:**
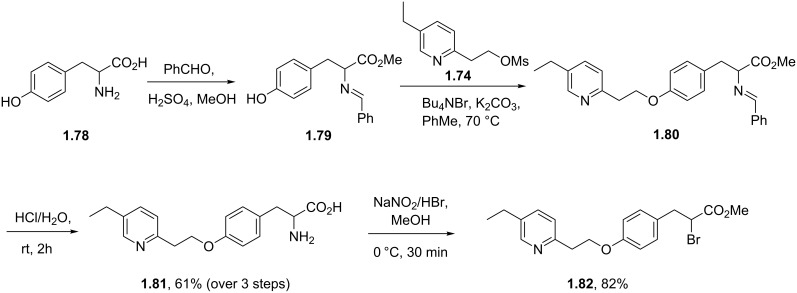
Route towards pioglitazone utilising tyrosine.

Alternatively, an equivalent acid functional material can be prepared starting from tyrosine (**1.78**) via a dual protection of the amino acid unit as the methyl ester and the amine as the benzaldehyde imine (Scheme 14). This is then followed by analogous ether formation with the previously generated mesylate **1.74**. Intermediate **1.80** is then hydrolysed to reveal once again the amino acid functionality, which upon diazotisation in the presence of hydrobromic acid selectively forms the α-bromo ester **1.82**.

**Scheme 15:**
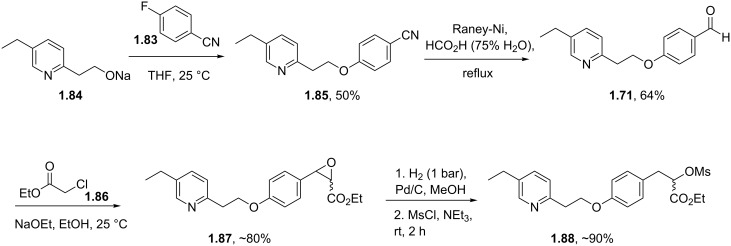
Route towards pioglitazone via Darzens ester formation.

A more direct S_N_Ar approach utilising 4-fluorobenzonitrile as the acceptor and the sodium alkoxide of hydroxyethylpyridine **1.84** as the nucleophile has been successfully conducted (Scheme 15) [49]. The nitrile unit is partially reduced and hydrolysed to the benzaldehyde **1.71** using Raney-Ni under transfer hydrogenation conditions in wet formic acid. A Darzens reaction between this aldehyde and ethyl chloroacetate in the presence of sodium ethoxide delivers epoxide **1.87**. The material is next subjected to hydrogenolysis using Pd/C in methanol with a 1 bar hydrogen pressure to reductively ring open the epoxide. Finally, the transformation of the alcohol to the mesylate **1.88** occurs under standard conditions.

**Scheme 16:**
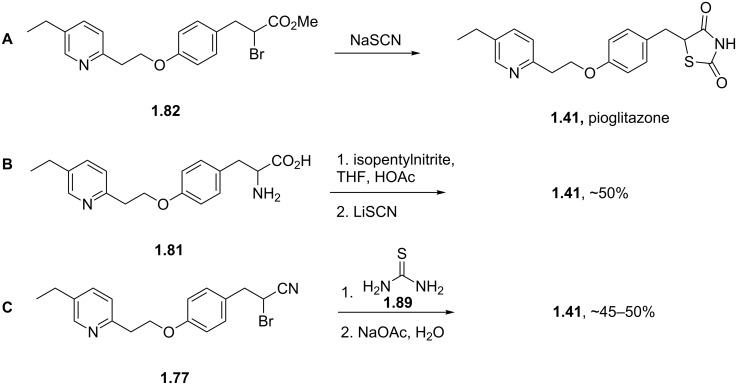
Syntheses of the thiazolidinedione moiety.

In order to complete the syntheses of pioglitazone as outlined in the previous schemes several procedures for the installation of the thiazolidinedione ring system have been reported [50]. For instance the α-bromoester of intermediate **1.82** will render the desired heterocycle upon treatment with sodium isothiocyanate (Scheme 16, A). Alternatively, the α-amino acid portion of **1.81** can be diazotised under standard conditions and will subsequently deliver the same product when treated with lithium isothiocyanate (Scheme 16, B). Finally, α-bromonitrile **1.77** can be condensed with thiourea to give pioglitazone after aqueous work-up (Scheme 16, C).

**Scheme 17:**
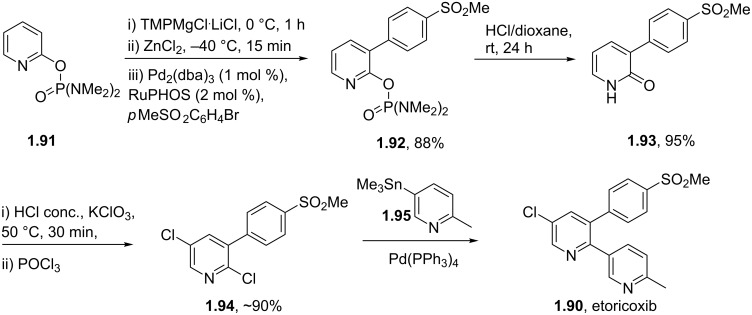
Synthesis of etoricoxib utilising Negishi and Stille cross-coupling reactions.

Etoricoxib (**1.90**) is a member of a new class of NSAID (non-steroidal anti-inflammatory drug) developed by Merck possessing a simple pyridine core [51]. It is reported to show a 160-fold selectivity for COX-2 over COX-1 (cyclooxygenase 1 and 2) and so it is hoped that it will not display long term side effects such as gastric ulceration, common to non-selective COX inhibitors. In many syntheses the presence of aryl–aryl linkages commands the use of Pd-mediated cross coupling reactions which dictates access to functionalised pyridines as starting materials. However, Knochel has recently reported an approach to forming the first aryl–aryl C–C bond by a directed lithiation of a pyridine **1.91** followed by conversion to its organozinc derivative. This intermediate then undergoes a high-yielding Negishi cross-coupling reaction with an arylbromide (Scheme 17) [52]. After acidic hydrolysis of the phosphoramidate directing group the resulting pyridone **1.93** is subjected to a sequence of chlorinations rendering a suitably functionalised coupling partner for a final Stille coupling to yield etoricoxib.

**Scheme 18:**
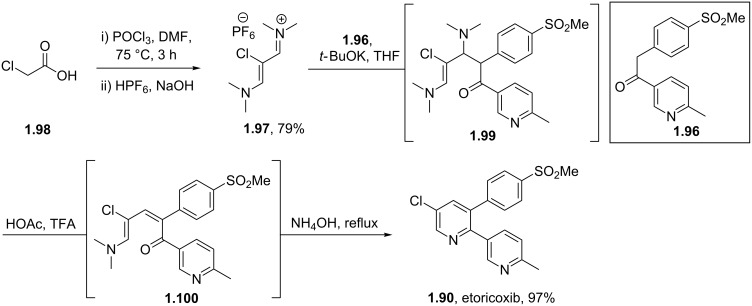
Synthesis of etoricoxib via vinamidinium condensation.

Although the synthesis of etoricoxib as depicted in Scheme 17 very efficiently forms the key aryl–aryl bonds and is suitable for analogue synthesis the route is not considered feasible for large scale synthesis due to the toxicity of the tin species and the inherent costs of the specifically functionalised starting materials and palladium catalysts. For this reason a de novo synthesis of the central pyridine ring was preferred [53]. Here, it was found, that the union of an enolisable ketone **1.70** with a vinamidinium species **1.97** reliably forms the desired heterocycle in excellent yield via a one pot procedure (Scheme 18). Moreover, the vinamidinium reagent can be formed in situ [54] and neatly introduces the required chloride substituent at the 5-position.

**Figure 2:**
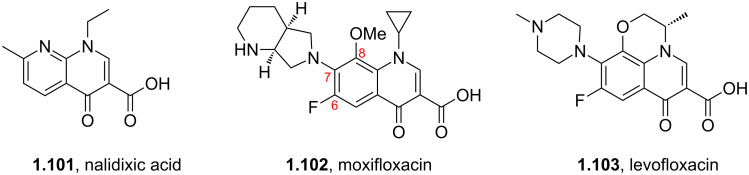
Structures of nalidixic acid, levofloxacin and moxifloxacin.

While isolated pyridines are a vital component in numerous drugs the related quinoline/quinolone scaffolds are becoming increasingly common. One such example is nalidixic acid (**1.101**, in fact a naphthyridone, Figure 2). Nalidixic acid is a prototype quinolone antibiotic that has been used extensively as an effective treatment against both gram positive and gram negative bacteria. In general, quinolone antibiotics act by interfering with the enzymes DNA-gyrase and/or topoisomerase of bacteria [55]. Moxifloxacin (**1.102**, Avelox) and levofloxacin (**1.103**, Levaquin) are two third-generation fluoroquinolone antibiotics which also appear amongst the top selling drugs. These compounds display similar SAR data [56]. For instance, in the case of moxifloxacin the fluorine atom in the C6 position enhances microbial activity while a methoxy group in the C8 position is reported to increase potency and decrease toxicity. Furthermore, it was found that a cyclopropyl group was beneficial for the enzyme–DNA binding complex while the bulky nitrogen-based appendage at C7 helps to bind to DNA gyrase and hinders the efflux of the drug from the bacterial cell.

**Scheme 19:**
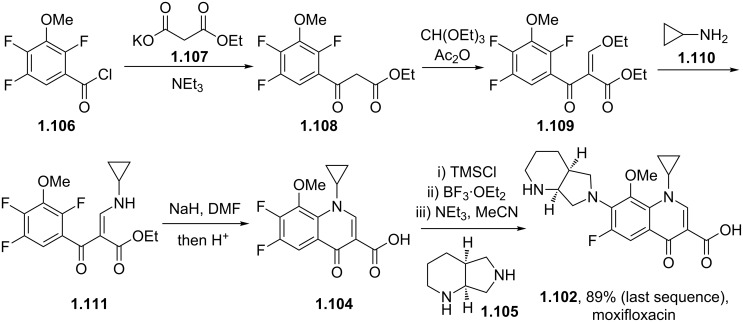
Synthesis of moxifloxacin.

Due to the elaborate substitution pattern of the parent quinolone ring systems these compounds are usually prepared via a linear consecutive sequence. In the case of moxifloxacin, an intramolecular base catalysed nucleophilic aromatic substitution is used to prepare the bicyclic ring system of the highly substituted aromatic **1.104** (Scheme 19). A S_N_Ar reaction is then used to introduce the saturated piperidinopyrrolidine appendage **1.105** to furnish the desired structure [57–60]. In order to obtain a high yield for the substitution reaction a one-pot procedure was developed, initial masking of the acid (**1.104**) is achieved by silylation with subsequent borane chelate formation. Addition of the amine nucleophile **1.105** under basic conditions then renders the desired product in high yield. The available patent literature however does not comment on regioselectivity issues of the S_N_Ar reaction due to the presence of the second fluoride substituent in the substrate, although not necessarily as electronically favourable for displacement it is certainly more accessible.

**Scheme 20:**
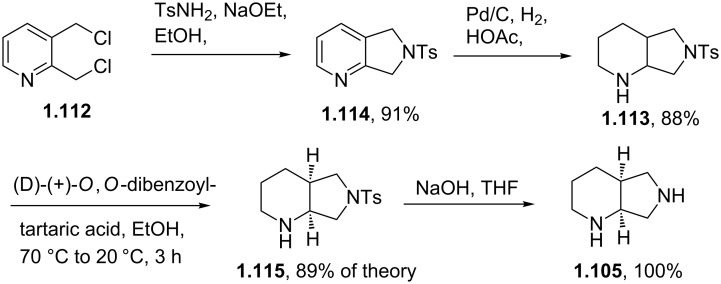
Synthesis of (*S*,*S*)-2,8-diazabicyclo[4.3.0]nonane **1.105**.

The saturated (*S*,*S*)-2,8-diazabicyclo[4.3.0]nonane (**1.105**) used in the final step can be prepared by a double nucleophilic substitution between tosylamine and 2,3-*bis*-chloromethylpyridine (**1.112**) followed by catalytic reduction of the resulting bicycle using palladium on carbon in acetic acid (Scheme 20). As the corresponding sulfonamide **1.113** was found to be a crystalline solid a resolution using (D)-(+)-*O*,*O*-dibenzoyltartaric acid was reported to separate the enantiomers [61].

**Scheme 21:**
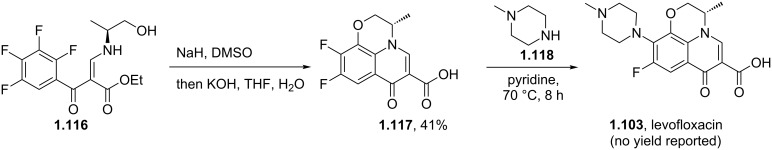
Synthesis of levofloxacin.

By analogy to moxifloxacin the synthesis of levofloxacin (**1.103**) can be accomplished using an intramolecular double S_N_Ar reaction sequence converting the tetrafluoroarene **1.116** into the required tricyclic fluoroquinolone product **1.117** (Scheme 21). A final intermolecular S_N_Ar reaction then introduces the methylpiperazine unit and thus completes the synthesis of this antibiotic compound [62].

**Scheme 22:**
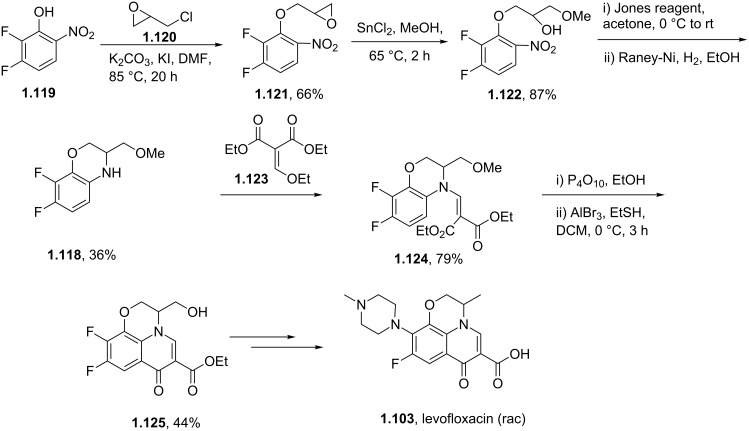
Alternative approach to the levofloxacin core **1.125**.

Alternatively, an elaborated benzoxazine substrate **1.118** may be prepared via another linear sequence starting from phenol **1.119**, which is first alkylated with epichlorohydrin (**1.20**) [63] (Scheme 22). The resulting epoxide **1.121** can then be ring opened with methanol in the presence of a tin Lewis acid yielding alcohol **1.122**, which when subjected to Jones-oxidation conditions and Raney-Ni-mediated hydrogenation furnishes in moderate yield the benzoxazine **1.118**; via the imine intermediate. This material can then be condensed with diethyl ethoxymethylenemalonate (**1.123**), and further cyclised via an intramolecular Friedel–Crafts acylation promoted by polyphosphate. Aluminium tribromide assisted demethylation of the pendant methoxy group of this tricyclic structure gives access to the corresponding alcohol **1.125** which is easily converted to the final structure of levofloxacin via a further four steps (i.e. introduction of the *N*-methylpiperazine unit via S_N_Ar reaction, activation and displacement of the primary alcohol and ester hydrolysis).

### 2. Dihydropyridines and piperidines

**Figure 3:**
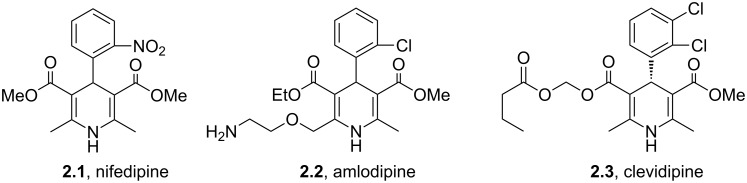
Structures of nifedipine, amlodipine and clevidipine.

The prevalence of dihydropyridines and their fully reduced counterparts the piperidines is high in many drug substances due to their high transport tolerance in many active processes and their structural spacing especially in a 1,4-disposition. The Hantzsch synthesis is the classical approach towards dihydropyridines and consequently has been utilised in a large number of drugs particularly those used as ion channel blockers. One of the earliest examples, nifedipine (**2.1**, Adalat, Figure 3) was introduced to the market in the 1970s as an antihypertensive and antianginal compound, reducing high blood pressure and addressing angina pectoris (acute chest pain) by increasing the blood flow to the heart. Various analogues of this early calcium ion channel blocker have been prepared. Amlodipine (**2.2**, Norvasc) from Pfizer which is of the same class is a superior long acting calcium channel blocker which possesses more desirable properties than nifedipine as it can be taken once daily as a tablet and comes with no dietary restrictions as well as eliciting less adverse side effects. The trend to base new ion channel blockers on the dihydropyridine scaffold continues even today with clevidipine (**2.3**, Cleviprex) which obtained FDA approval in 2008. In contrast to the previous compounds clevidipine is a very short acting calcium channel blocker and is used as a single enantiomer being administered intravenously rather than orally. Due to its labile hydroxymethylene ester moiety clevidipine is readily hydrolysed by serum esterases leading to rapid therapeutic action and subsequent clearance as well as displaying very little renal or hepatical side effects.

**Scheme 23:**
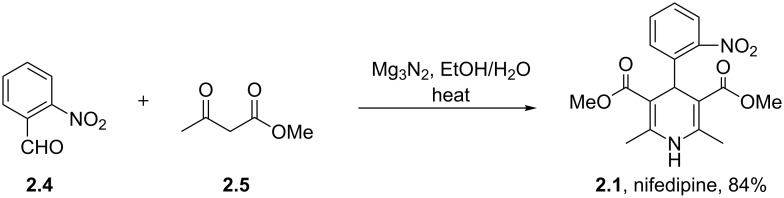
Mg_3_N_2_-mediated synthesis of nifedipine.

The synthesis of nifedipine can be readily achieved using the classical Hantzsch reaction condensing 2-nitrobenzaldehyde (**2.4**) with methyl acetoacetate (**2.5**) in the presence of an ammonia source. However, gaseous ammonia as well as ammonia solutions are considered difficult reagents due to handling issues, often giving problems due to corrosion as well as the obvious associated stench. One interesting protocol circumventing the problem describes the use of bench stable magnesium nitride, a solid material which upon hydrolysis liberates the required ammonia in situ (Scheme 23) [64]. However, there have been several reports on explosions caused by runaway reactions so extreme care must be administrated when using this reagent [65].

**Scheme 24:**
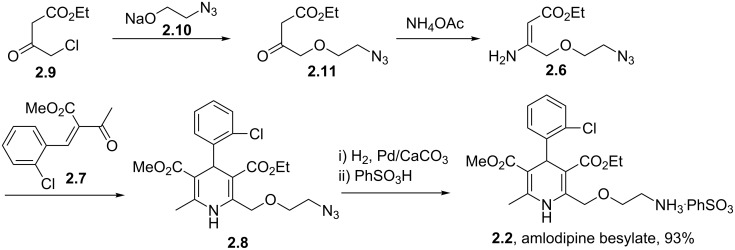
Synthesis of *rac*-amlodipine as besylate salt.

From a qualitative comparison of the structures of nifedipine, amlodipine and clevidipine it becomes obvious that the non-symmetrical nature of structures **2.2** and **2.3** impose additional challenges to their synthesis routes. Amlodipine’s (**2.2**) synthesis requires a more linear approach to install the different ester groups as well as the aminoethanol appendage. In a patented route to this compound the assembly is realised using a stepwise condensation between enamine **2.6**, which already contains the masked aminoethanol unit, and the advanced Michael acceptor **2.7** delivering dihydropyridine **2.8**. Mild azide reduction and subsequent formation of the besylate salt renders the final formulation of amlodipine (Scheme 24) [66]. The besylate salt of amlodipine has been introduced to the market as the racemate, nevertheless, separation of both enantiomers on semi-preparative scale has been demonstrated allowing biological evaluation of either enantiomer and comparison of the obtained results. Through these studies it was found that the *S*-(−)-enantiomer of amlodipine is the biologically active one although there appears to be no significant difference in the pharmacokinetic behaviour between the *R*-(+) form, the *S*-(−) form or the racemate leading to the conclusion that an enantioselective synthesis leading to solely *S*-(−)-amlodipine would be beneficial only if economically feasible [67].

**Scheme 25:**
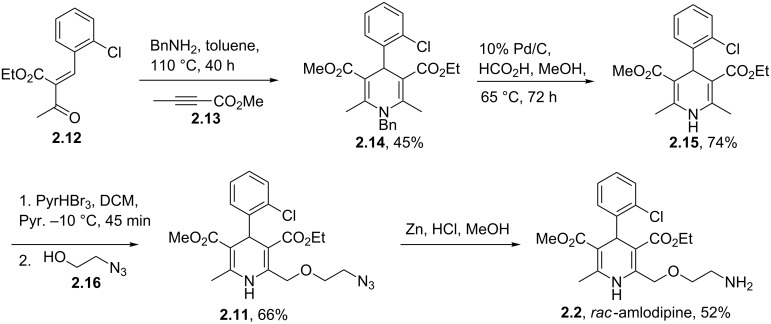
Aza Diels–Alder approach towards amlodipine.

An alternative strategy to this molecule begins with an interesting aza-Diels–Alder reaction between the Knoevenagel adduct **2.12** and methyl butynoate **2.13** (Scheme 25) [68]. The reaction is reported to progress in high regioselectivity (>50:1) albeit in moderate isolated yield. Following debenzylation of the Diels–Alder product a selective allylic bromination was achieved again in good selectivity (>80:20, mono:di) using a buffered solution of pyridinium perbromide in methylene chloride/pyridine at low temperature. After purification of the resulting intermediate by flash column chromatography the direct displacement of the newly installed bromide by an azidoethanol moiety yields the desymmetrised dihydropyridine. This regioselective bromination reaction was studied in some detail by the Warner–Lambert Company and is reported to give superior outcomes when compared to the direct Hantzsch synthesis of non-symmetrical dihydropyridines with respect to product yield (70–90% vs 10–30%) and ease of purification [69]. Finally, zinc-mediated reduction of the azide furnishes racemic amlodipine (**2.2**).

**Scheme 26:**
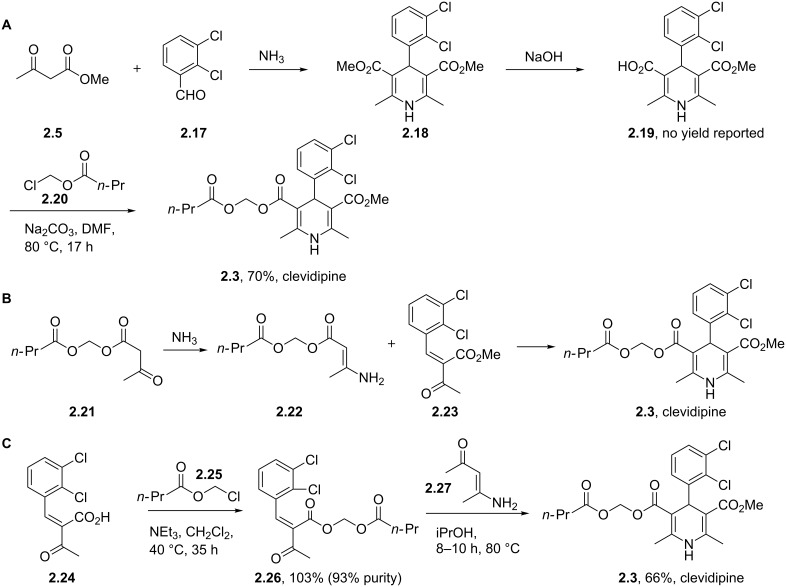
Routes towards clevidipine.

The last mentioned dihydropyridine based pharmaceutical is clevidipine (**2.3**), a third generation calcium channel blocker used primarily to rapidly decrease and stabilise blood pressure following cardiac surgery. The presence of the readily hydrolysed acyloxyl methyl ester accounts for much of its enhanced potency acting as a rapid response vasodilator. It is reported that both enantiomers undergo esterase-mediated hydrolysis with short half-lifes of around two minutes and possessing similar medical and physiological profiles, consequently this means clevidipine can be safely administered in its racemic form [70]. There are two main routes described to this structure (**2.3**, Scheme 26): A standard Hantzsch synthesis between 2,3-dichlorobenzaldehyde (**2.17**), ammonia and methyl acetoacetate (**2.5**) furnishes the symmetric dihydropyridine **2.18**, which can be selectively mono-saponified and the resulting carboxylic acid group alkylated by treatment with chloromethyl butyrate (**2.20**) (Route A). Unfortunately, for this apparently straightforward synthesis no commentary regarding why the initial hydrolysis was so selective was made and the conditions used do not readily contest to the observed selectivity. However, this route was not adopted, instead a second more structured synthesis was eventually pursued (Scheme 26; Route B). Thus, 2,3-dichlorobenzaldehyde (**2.17**) was treated with methyl acetoacetate (**2.5**) resulting in the formation of enone **2.23**, this material was subsequently condensed with enamide **2.22** to directly yield clevidipine (**2.3**, Route B, [71]). A final strategy is depicted in Scheme 26 (Route C, [72]). This approach is similar to route B, however, it introduces the amine as part of the readily available 3-aminocrotonate (**2.27**) which subsequently undergoes the Hantzsch reaction with the elaborate Michael acceptor **2.26**.

**Figure 4:**
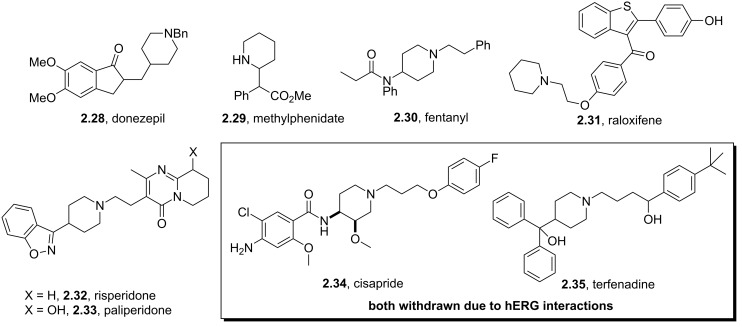
Examples of piperidine containing drugs.

The fully reduced pyridine unit, the piperidine ring system, is one of the most frequently encountered heterocycles found in pharmaceutical agents, typically acting either as a linker or in order to improve the drugs pharmacokinetic profile (increased solubility under physiological conditions and reduced logD value). Such heterocyclic systems are often disubstituted at the 1,2- or 1,4-positions resulting in easy access from the corresponding pyridone or piperidone precursors. Consequently, such substructures feature in numerous market drugs like donepezil (**2.28**), methylphenidate (**2.29**), fentanyl (**2.30**), raloxifene (**2.31**), risperidone (**2.32**) and paliperidone (**2.33**, Figure 4). Interestingly, a number of piperidine containing drugs (e.g. cisapride (**2.34**), terfenadine (**2.35**)) have been found to interact with hERG potassium ion channels resulting in a cardiac arrhythmia that can lead to fainting or even sudden death. In most of these cases this rare but severe side reaction cannot be tolerated and has therefore lead to drug failure in preclinical trials or even withdrawals of marketed drugs (e.g. cisapride, terfenadine or astemizole). It therefore can be stated that careful balancing is essential when incorporating basic sites into drug molecules in order to avoid off-target binding to hERG channels. Structural data now available on these hERG channels show the presence of two aromatic amino acid residues, Tyr652 and Phe656, that are located in an exposed area within the S6 domain pointing towards the possibility of cation π-interactions explaining the affinity of drugs containing tertiary amines towards hERG channels. Consequently, careful and early-stage examination of hERG affinity is nowadays a vital part of assessing preclinical risks of new drug candidates [73].

The piperidine unit is also incorporated into drugs in order to fulfil more significant tasks, i.e. being part of the pharmacophore itself or providing extended H-bonding networks for improved binding affinities. Furthermore, stereochemical issues encountered when the piperidine ring comprises a part of more strained bridged bicyclic system or a polycyclic scaffold are becoming more common. In the next section selected examples of these newer piperidine architectures will be discussed.

**Figure 5:**
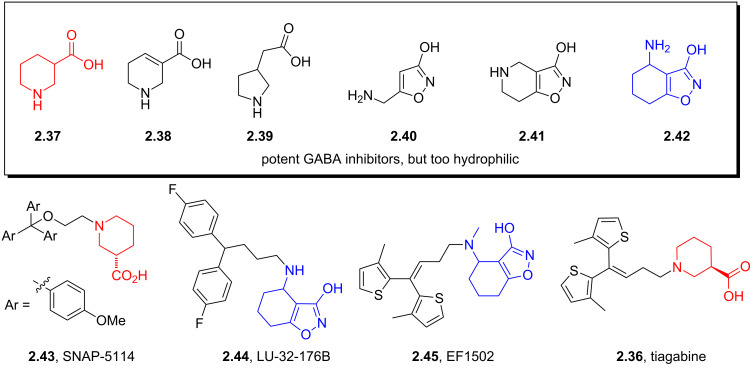
Discovery of tiagabine based on early leads.

Tiagabine (**2.36**) is a GABA uptake inhibitor originally developed over 20 years ago by Novo Nordisk and Abbott and is currently used as anticonvulsant in the treatment of epilepsy. For a long time it has been recognised that the simple core alkaloid structures as found in nipecotic acid (**2.37**), guvacine (**2.38**), β-homoproline (**2.39**), muscimol (**2.40**), THPO (**2.41**) or exo-THPO (**2.42**, Figure 5) are potent inhibitors of GABA receptors, however, their hydrophilic nature meant that they did not cross the lipophilic blood-brain barrier. Therefore additional non-polar biaryl-appendages have been attached to these motifs separated by a short alkyl chain to moderate their bioavailability resulting in the ultimate discovery of tiagabine (**2.36**) which has an embedded (*R*)-nipecotate unit as the class indicative pharmacophore.

**Scheme 27:**
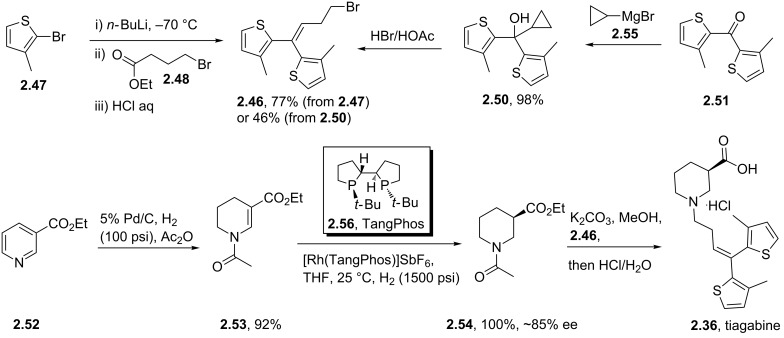
Synthetic sequences to tiagabine.

The synthesis of tiagabine (**2.36**) is readily accomplished by the union of ethyl nipecotate with the *bis*-thiophene containing fragment **2.46**, which itself can be accessed by various methods (Scheme 27) [74]. For instance, 2-bromo-3-methylthiophene (**2.47**) can be lithium-halogen exchanged using *n*-butyllithium. Two equivalents of the resulting anion are reacted with ethyl bromobutyrate **2.48** to give the unsaturated alkene subunit **2.46** upon elimination of water (Scheme 27, path A). Alternatively, the same intermediate (**2.46**) can be obtained by an ingenious acid catalysed ring opening of the cyclopropane derivate **2.50**, which is readily generated from Grignard-addition to the *bis*-thiophenyl ketone **2.51** (Scheme 27, path B). The required (*R*)-enantiomer of ethyl nipecotate can in turn be obtained by a number of different methods including the resolution of the racemate using L-(+)-tartaric acid obtained from full saturation of ethyl nicotinate. More modern methods involve a two-step process wherein ethyl nicotinate (**2.52**) is hydrogenated to the vinylogous carbamate **2.53** in the presence of acetic anhydride. Then the intermediate **2.53** can be subjected to an asymmetric hydrogenation utilising rhodium-based catalyst systems at elevated hydrogen pressures rendering the desired ethyl (*R*)-nipecotate **2.54** [75]. Uniting the two fragments is affected by nucleophilic substitution of the homoallylic bromide **2.46** by the nucleophilic ethyl (*R*)-nipecotate yielding tiagabine (**2.54**) after ester hydrolysis. Isolation of the final API requires careful adjustment of the pH using HCl to ensure isolation of the correct hydrochloric salt polymorph.

**Figure 6:**
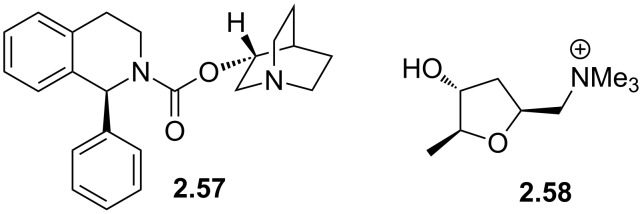
Structures of solifenacin (**2.57**) and muscarine (**2.58**).

The piperidine scaffold also features in a recently discovered pharmaceutical, namely solifenacin (**2.57**, Vesicare), a competitive antagonist of the muscarinic acetylcholine receptor used in the treatment of an overactive bladder. This species was co-developed by Astellas and GSK scientists and consists of a chiral hydroisoquinoline linked to a (*R*)-quinuclidinol unit through a carbamate linkage (Figure 6). Upon protonation the tertiary amine of the quinuclidine is expected to resemble the ammonium substructure of muscarine (**2.58**) [76].

**Scheme 28:**
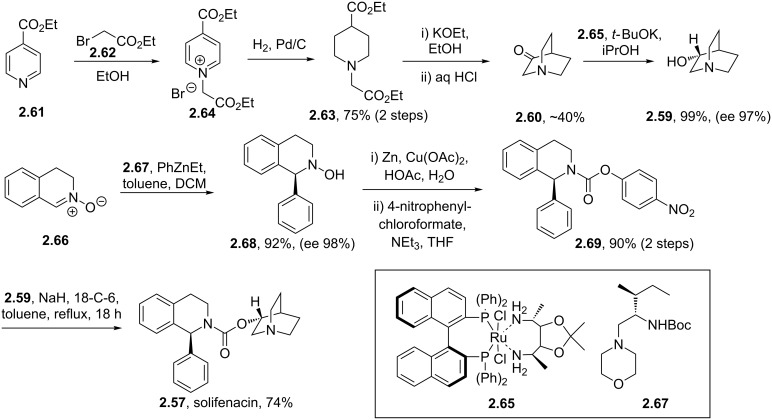
Enantioselective synthesis of solifenacin.

This molecule can be prepared by direct coupling of the (*R*)-quinuclidinol and tetrahydroisoquinoline carbamate partner (Scheme 28). The (*R*)-quinuclidinol (**2.59**) itself can be accessed from quinuclidone (**2.60**), and is most conveniently prepared by alkylation of ethyl isonicotinate (**2.61**) with ethyl bromoacetate (**2.62**) followed by full reduction of the pyridine ring therefore yielding the corresponding piperidine **2.63**. A base-mediated Dieckmann cyclisation and Krapcho decarboxylation [77] then furnishes **2.60**. Traditionally, the reduction of **2.60** to prepare **2.59** can be carried out under fairly mild hydrogenation conditions that ultimately produce racemic quinuclidinol. However, an improved approach makes use of a Noyori-type asymmetric reduction employing a BINAP ligated RuCl_2_ and a chiral diamine to yield the desired (*R*)-quinuclidine in high yield and enantioselectivity [78].

The enantioselective synthesis of the tetrahydroisoquinoline fragment is achieved via an asymmetric addition of phenylethylzinc to the imine *N*-oxide **2.66** yielding the corresponding 3,4-dihydroisoquinoline-*N*-hydroxide **2.68**. Further reductive cleavage of the hydroxylamine moiety followed by activation with 4-nitrophenyl chloroformate [79] yields the intermediate **2.69**. In the last step of the sequence the addition of (*R*)-quinuclidinol generates solifenacin (**2.57**).

**Figure 7:**
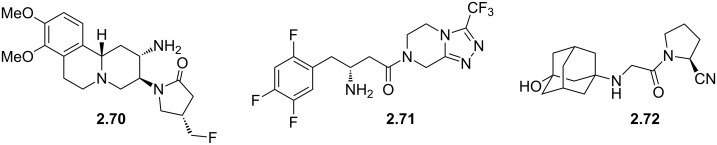
Structures of DPP-4 inhibitors of the gliptin-type.

**Scheme 29:**
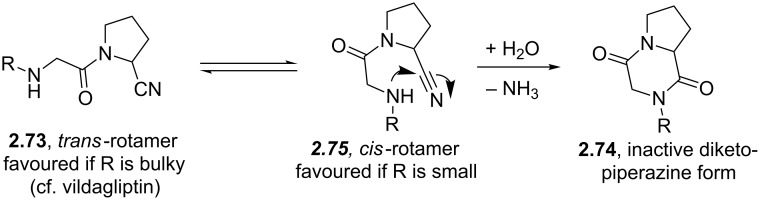
Formation of inactive diketopiperazines from cis-rotameric precursors.

Carmegliptin (**2.70**) is an anti-diabetes drug which is currently in late stage clinical trials. It represents a further structural advancement from the other existing marketed drugs in this class, sitagliptin (**2.71**, Januvia) and vildagliptin (**2.72**, Zomelis, Figure 7). These compounds are all members of the dipeptidyl peptidase 4 class (DPP-4), a transmembrane protein that is responsible for the degradation of incretins; hormones which up-regulate the concentration of insulin excreted in a cell. As DPP-4 specifically cleaves at proline residues, it is unsurprising that the members of this drug class exhibit an embedded pyrrolidine ring (or mimic) and additional decoration (a nitrile or fluorinated alkyl substituent is present in order to reach into a local lipophilic pocket). One specific structural liability of the 2-cyano-*N*-acylpyrrolidinyl motif (**2.73**) is its inherent susceptibility towards diketopiperazine formation (**2.74**, Scheme 29) [80], however, one way to inhibit this transformation is to position a bulky substituent on the secondary amine nucleophile as is the case in vildagliptine (**2.72**).

**Figure 8:**
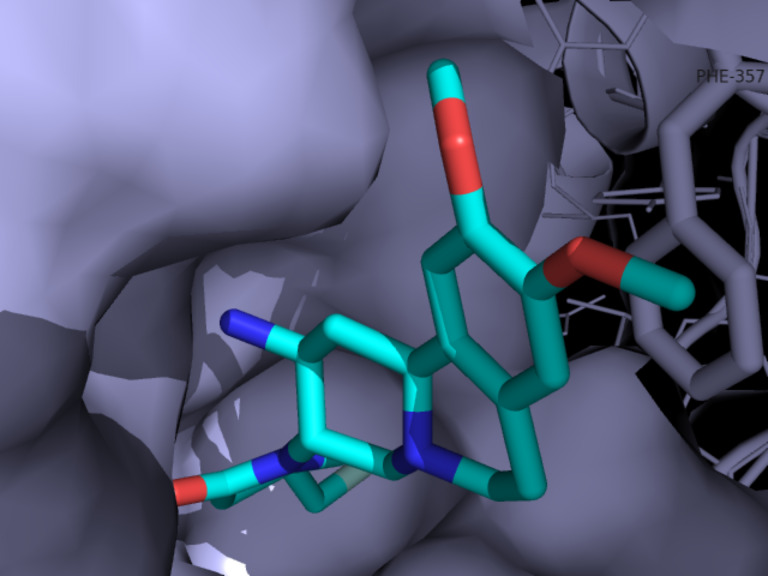
Co-crystal structure of carmegliptin bound in the human DPP-4 active site (PDB 3kwf).

A single crystal X-ray structure of carmegliptin bound in the human DPP-4 active site has been published indicating how the fluoromethylpyrrolidone moiety extends into an adjacent lipophilic pocket [81]. Additional binding is provided by π–π interaction between the aromatic substructure and an adjacent phenylalanine residue as well as through several H-bonds facilitated by the adjacent polar substituents (Figure 8).

**Scheme 30:**
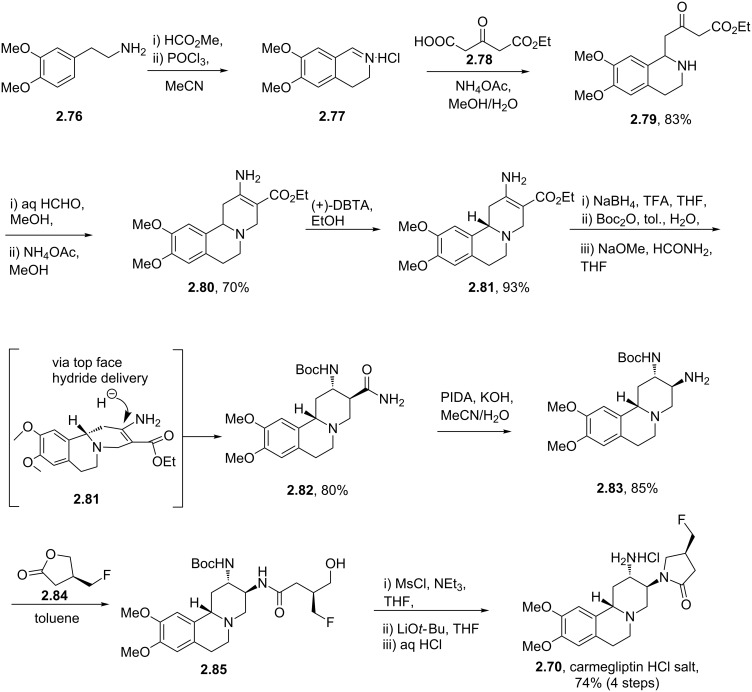
Improved route to carmegliptin.

The reported synthesis of carmegliptin enlists a Bischler-Napieralski reaction utilising the primary amine **2.76** and methyl formate to yield the initial dihydroquinoline **2.77** as its HCl salt (Scheme 30) [82]. This compound was next treated with 3-oxoglutaric acid mono ethyl ester (**2.78**) in the presence of sodium acetate. Decarboxylation then yields the resulting aminoester **2.79** which was progressed through an intramolecular Mannich-type transformation using aqueous formaldehyde to allow isolation of enaminoester **2.80** after treatment of the intermediate with ammonium acetate in methanol. The next step involves a very efficient crystallisation-induced dynamic resolution of the racemic material using the non-natural (*S*,*S*)-dibenzoyl-D-tartaric acid ((+)-DBTA). It is described that the desired (*S*)-enantiomer of compound **2.81** can be isolated in greater than 99% ee and 93% overall yield. This approach is certainly superior to the original separation of the two enantiomers (at the stage of the final product) by preparative chiral HPLC that was used in the discovery route (albeit it should be noted that both enantiomers were required for physiological profiling at the discovery stage). Next, a 1,2-*syn* diastereoselective reduction of enaminoester **2.81** occurs with high diastereocontrol imposed by the convexed presentation of the substrate for the formal conjugate addition and subsequent protonation steps. This is followed by Boc-protection and interconversion of the ethyl ester to its amide derivative **2.82** in 80% overall yield for this telescoped process. The primary amide in **2.82** was then oxidised via a modern variant of the classical Hoffmann rearrangement using phenyliodine diacetate (PIDA). Following extensive investigation it was found that slowly adding this reagent in a mixture of acetonitrile/water to a suspension of amide **2.82** and KOH gave clean conversion to the amine product in high yield. This new procedure was also readily scalable offering a cleaner, safer and more reliable transformation when compared to other related rearrangement reactions. During a further telescoped procedure amine **2.83** was treated with lactone **2.84** to regenerate the corresponding lactam after mesylate formation. Finally, removal of the Boc-group with aqueous hydrochloric acid furnished carmegliptin as its HCl salt.

### 3. Pyrimidines and quinazolines

Whilst pyridine rings and their partially or fully saturated derivatives are very frequent components of pharmaceutical species there are also a considerable number of compounds based on diazine and triazine ring systems. Amongst the diazines, pyrimidine-derived analogues (1,3-diazines) are the most common. Pyridazines (1,2-diazines) and pyrazines (1,4-diazines) are less prominent. The main reasons are most probably that pyrimidines are highly abundant in important biogenic molecules such as the ribonucleotides and that this heterocycle can be readily obtained through well-established condensation chemistries.

In the next sections the most common syntheses of a number of drugs containing the pyrimidine as well as pyridazine and pyrazine scaffolds are presented. Furthermore, some non-aromatic derivatives including bicyclic analogues of these structures will be introduced.

The additional nitrogen atom in pyrimidine leads to a significantly reduced basicity when compared to simple pyridine (p*K*_a_[pyrimidine] = 1.1; p*K*_a_[pyridine] = 5.3) as well as a much more electron-deficient ring system. However, owing to the presence of two nitrogen atoms pyrimidines form very tight hydrogen-bonding arrays as seen in DNA and RNA. Synthetically, the electron-poor nature of pyrimidines accounts for the manifold functionalisation pathways using nucleophilic aromatic substitution chemistry. Electrophilic aromatic substitution reactions are more common during the preparation of substituted pyridines.

**Figure 9:**
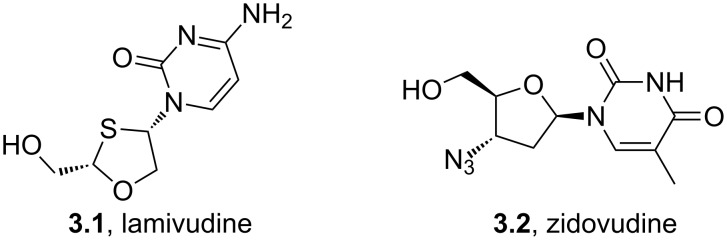
Structures of lamivudine and zidovudine.

Many antiviral agents show a close structural resemblance to the nucleotides uracil, thymine and cytosine. Lamivudine (**3.1**) or zidovudine (**3.2**) are typical examples of pyrimidines/pyrimidones bound to a ribose-analogue (Figure 9) [83].

**Scheme 31:**
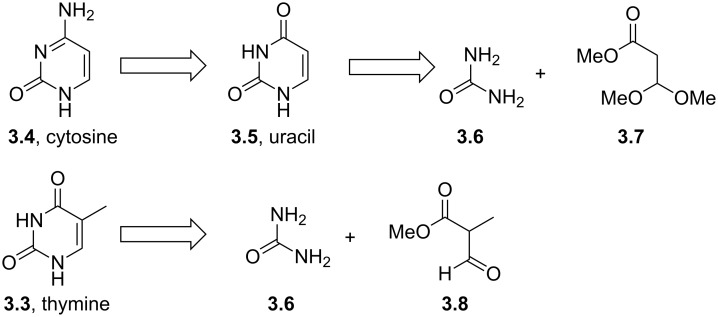
Typical routes accessing uracil, thymine and cytosine.

These molecules function by terminating biosynthesis of nucleic acids eventually interrupting the division of virally infected cells. Most commonly the pyrimidine/pyrimidone substructure has been introduced as a preassembled unit. Thymine (**3.3**), cytosine (**3.4**) and uracil (**3.5**) are all industrially accessed on large scale utilising various robust routes (Scheme 31) [84–85]. Most of these transformations use condensation reactions between urea (**3.6**) and a *bis*-electrophile to generate the desired structures. For instance, ester **3.7** furnishes uracil (**3.5**), which in turn can be converted into cytosine (**3.4**) by selective amination with ammonia/ammonium chloride (Scheme 31). Alternatively, thymine (**3.3**) may be accessed by reacting methyl formylpropionate (**3.8**) which acts as a *bis*-electrophile with urea.

**Scheme 32:**
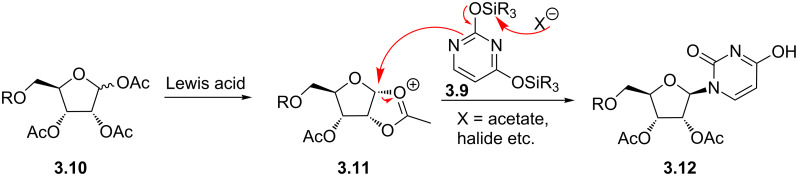
Coupling between pyrimidones and riboses via the Vorbrüggen nucleosidation.

Following construction of the pyrimidone core the linkage to the anomeric position of a ribose is best achieved using the Vorbrüggen nucleosidation (Scheme 32) [86]. In this procedure an *O*-silylated pyrimidone (**3.9**) is combined with the acylated or benzoylated ribose derivative **3.10** in the presence of a strong Lewis acid. The generation of the reactive oxycarbenium ion as well as the liberated nucleophilic pyrimidone therefore occurs in situ and allows for good stereochemical control.

**Scheme 33:**
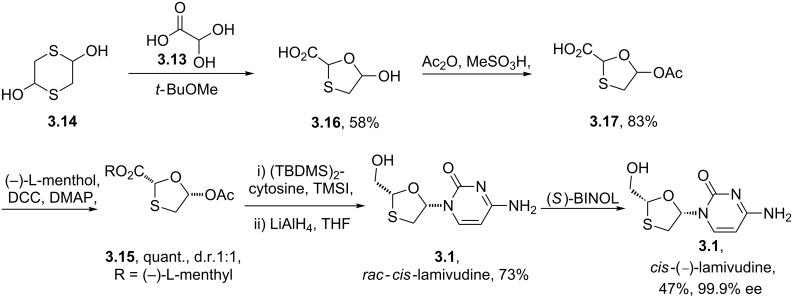
Synthesis of lamivudine.

The ribose component in these drugs is either derived directly from ribose itself as in zidovudine or prepared by total synthesis as in the case of lamivudine. The oxathiolane ring in lamivudine for instance can be prepared via an acetal exchange reaction between glyoxilic acid monohydrate (**3.13**) and 1,4-dithiane-2,5-diol (**3.14**) (Scheme 33) [87]. Subsequent acetylation of the hydroxy group followed by ester formation using (−)-L-menthol permits crystallisation separation of the two diastereoisomers. The use of *bis*-TBDMS-cytosine as a coupling partner in the presence of trimethylsilyl iodide together with oxathiolane **3.15** produces lamivudine (**3.1**) after reduction of the menthyl ester.

Interestingly, the *cis*-(−)-enantiomer of structure **3.1** was reported to be much less cytotoxic although both *cis*-isomers are independently active against HIV. Recently, a protocol for resolving the racemate on large scale using (*S*)-BINOL was reported [88]. This method works via the co-crystal formation between (*S*)-BINOL and *cis*-(−)-lamivudine forming a binary complex which was characterised by single crystal X-ray crystallography.

**Scheme 34:**
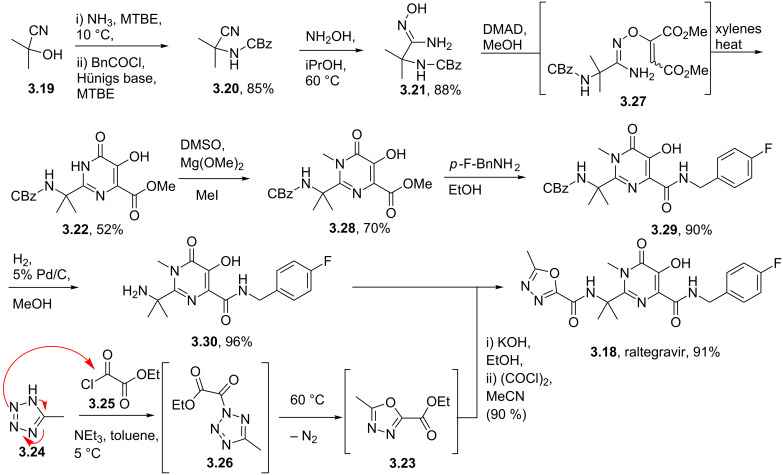
Synthesis of raltegravir.

Raltegravir (**3.18**, Isentress), is another important pyrimidine-based anti-HIV drug which was launched by Merck in 2008 [89]. Structurally, this HIV-integrase inhibitor consists of a fully substituted pyrimidone core flanked by an oxadiazole ring as well as an additional terminal *para*-fluorobenzyl unit. The central pyrimidone core was accessed in a linear fashion starting by amination of acetone cyanohydrin (**3.19**) followed by CBz-protection of the resulting amine and finally aminolysis of the nitrile **3.20** with hydroxylamine [90] (Scheme 34). The resulting amidoxime **3.21** was then reacted with dimethylacetylene dicarboxylate (DMAD) which upon heating in xylenes undergoes ring-closure yielding the desired pyrimidine **3.22** but unfortunately only in modest yield. However, this key intermediate could be readily *N*-methylated, and in a simple extension to the sequence subjected to direct amide formation, debenzylation and finally coupled with the corresponding acid chloride of oxadiazole derivative **3.23**. This particular oxadiazole **3.23** was prepared via a clever sequence involving acylation of methyl tetrazole (**3.24**) with ethyl oxalylchloride (**3.25**) to form intermediate **3.26** which when heated extrudes nitrogen gas and subsequently collapses to the desired oxadiazole **3.23**, all progressing in high yield.

**Scheme 35:**
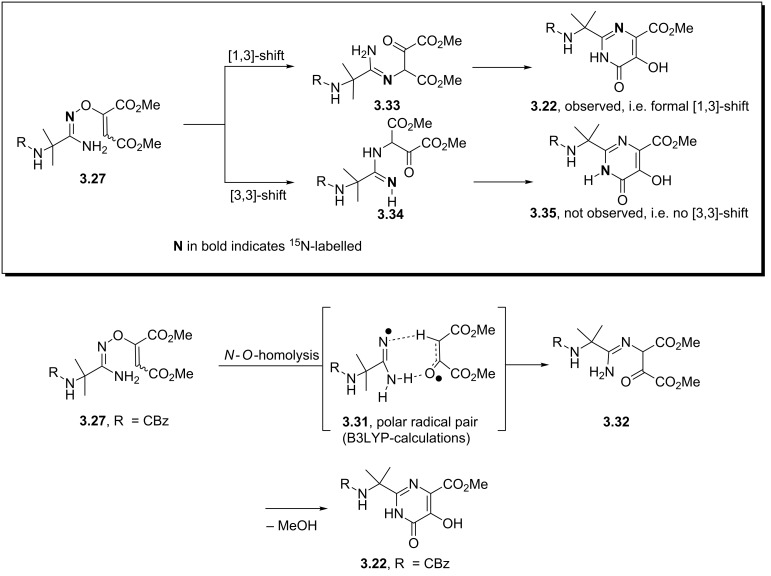
Mechanistic studies on the formation of **3.22**.

The formation of the hydroxypyrimidone core (**3.22**) of raltegravir deserves further discussion as its unexpected mechanism was only recently fully elucidated in a joint effort between Merck process chemists and the Houk group at UCLA [91]. These studies combined B3LYP density functional theory with labelling studies and revealed that the most likely pathway involves the formation of a tightly bound polar radical pair **3.31** resulting from thermal homolysis of the N–O bond (Scheme 35). This species subsequently recombines under formation of a C–N bond and a C=O double bond (**3.32**) allowing for the final cyclocondensation to occur with liberation of methanol. Furthermore these studies were able to disprove a potential alternative [3,3]-sigmatropic rearrangement step by incorporating ^15^N enriched precursors leading to the formation of pyrimidone **3.22**, which is only consistent with a formal [1,3]-sigmatropic rearrangement. Subsequent calculations demonstrated the high energy barrier for such a concerted [1,3]-shift, ultimately leading to the finding of the before-mentioned polar radical pair pathway which is about 8 kcal/mol lower in energy. This is consistent with the experimentally observed rate acceleration in case of the *Z*-isomer of **3.33** over the *E*-isomer which was also confirmed by calculations showing an energy gap of 3 kcal/mol.

**Figure 10:**
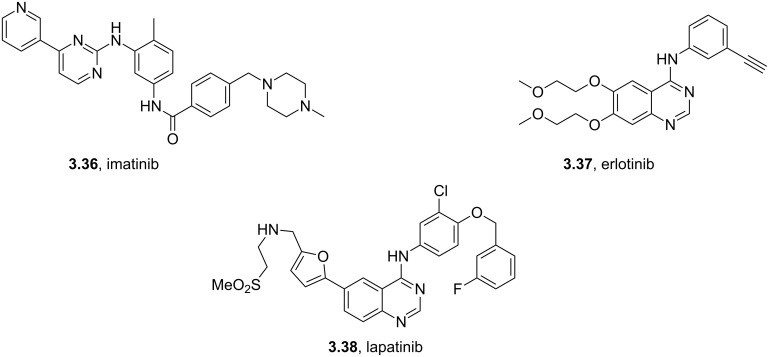
Structures of selected pyrimidine containing drugs.

In general the pyrimidine ring is well represented in a number of kinase inhibitors such as imatinib (**3.36**), erlotinib (**3.37**) and lapatinib (**3.38**) (Figure 10).

**Scheme 36:**
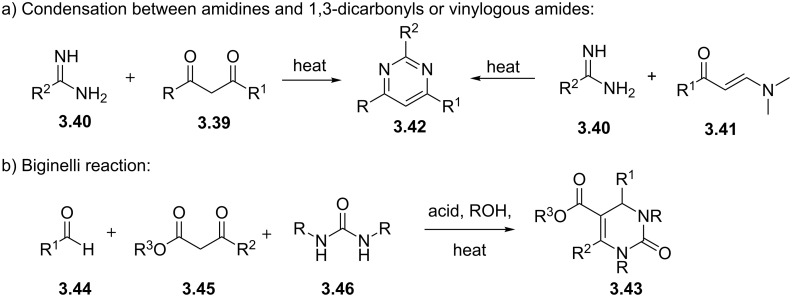
General preparation of pyrimidines and dihydropyrimidones.

In order to prepare the core heterocyclic unit a direct condensation between a 1,3-dicarbonyl compound **3.39** and an amidine or guanidine **3.40** is frequently employed (Scheme 36a). Alternatively, an amidine can be condensed with a vinylogous amide **3.41** resulting in the direct formation of 2,4-disubstituted pyrimidines. These condensations often require relatively harsh reaction conditions despite this they are of great value as they involve cheap or easily accessible materials and typically only form water as the principle byproduct.

In other syntheses the Biginelli reaction [92] has been employed to give rapid access to several related dihydropyrimidone structures **3.43** that can be easily oxidised to the corresponding aromatic forms. In this classic three-component reaction an aldehyde (**3.44**), an α-ketoester (**3.45**) and an urea (**3.46**) undergo a multicomponent coupling furnishing the desired heterocycle with up to five variable substituents (Scheme 36b). Consequently, these MCR (multicomponent reactions) are important in accessing highly functionalised building blocks commonly used in early research and discovery programs to enable scoping of structure activity patterns.

**Scheme 37:**
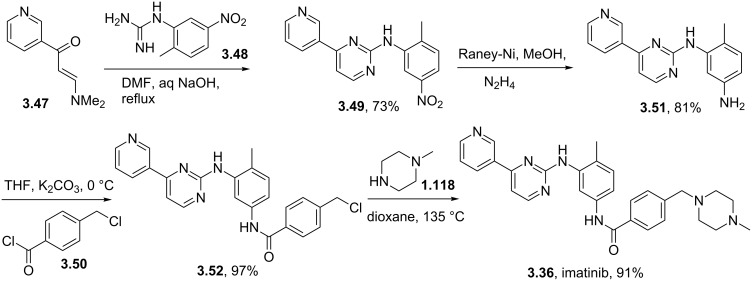
Synthesis of imatinib.

A modification of the above pyrimidine synthesis has been applied in the generation of imatinib (**3.36**, Gleevec) which is Novartis’ tyrosine kinase inhibitor used for the treatment of chronic myeloic leukaemia. In a patented route the aldol product **3.47** undergoes a condensation reaction with guanidine **3.48** in basic media to give the 2-aminopyrimidine **3.49** (Scheme 37) [93]. After generating the functional pyrimidine core a hydrazine-mediated reduction of the nitro group in the side chain was conducted with Raney-Nickel as the catalyst. Amide formation with 4-chloromethylbenzoyl chloride (**3.50**) and a direct displacement of the benzylic chloride with *N*-methylpiperazine (**1.118**) complete this synthesis of imatinib in excellent overall yields.

**Scheme 38:**
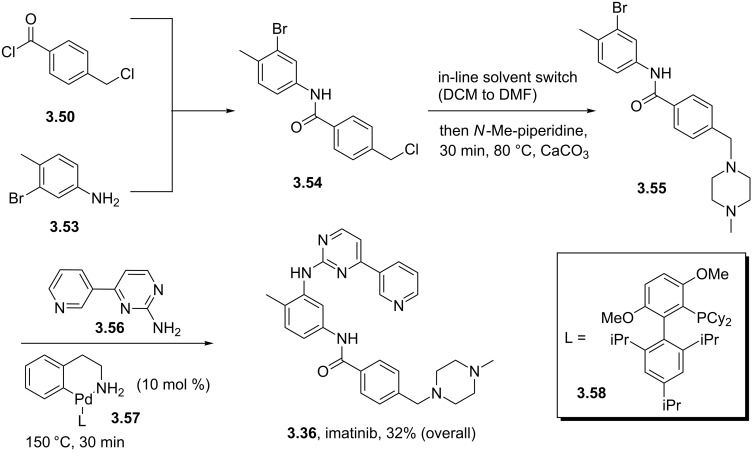
Flow synthesis of imatinib.

One noteworthy feature of this imatinib synthesis is that it is specifically designed for facile isolation of intermediates by precipitation due to their limited solubility in non-polar solvents [94]. Whilst this process was efficient in enabling the isolation of pure material after each step, it does not encourage telescoping of steps, which would in principal increase the overall efficiency of the process. Recently, similar approaches have been utilised in the academic environment using enabling techniques in a route to imatinib. For instance, our group has employed continuous flow synthesis methods to imatinib [95–96]. The route not only afforded imatinib but led to many previously inaccessible derivatives in an automated fashion within a single working day (Scheme 38). In addition, this particular sequence showcases the uses of scavenger resins for in-line purification as the synthesis progresses and features the use of a Buchwald–Hartwig amination in a late stage fragment coupling. While it was sufficient to access only small amounts of these structures (around 50 mg), these techniques are currently being adopted by several major pharmaceutical companies in order to enhance drug development and even manufacturing sequences.

**Scheme 39:**
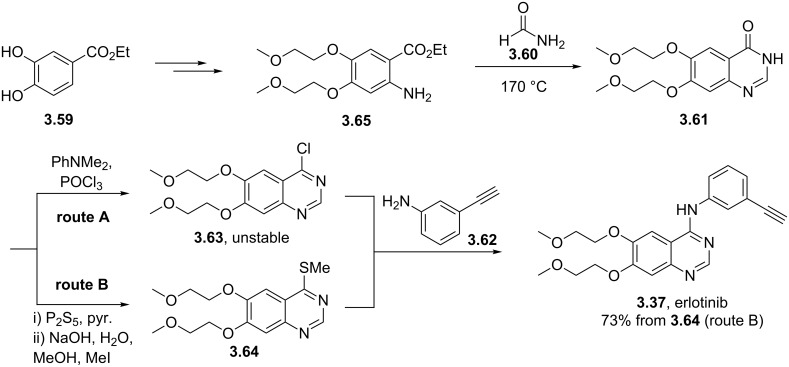
Syntheses of erlotinib.

Erlotinib (**3.37**, Tarceva) is another related medication which is used to treat non-small lung cancer as well as pancreatic cancer by targeting the epidermal growth factor receptor-tyrosine kinase. The structure feature of this compound is the trisubstituted quinazoline core bearing an aniline moiety and two polyether appendages (Scheme 39). A representative route to **3.37** [97–98] enlists ethyl 3,4-dihydroxybenzoate (**3.59**) which after double etherification and incorporation of an amine was then subjected to a condensation reaction with formamide (**3.60**) at elevated temperature. Chlorination of the resulting quinazolinone **3.61** with phosphorous oxychloride in the presence of *N*,*N*-dimethylaniline ultimately generates the starting material for the final S_N_Ar reaction with 3-aminophenylacetylene (**3.62**) and completes the optimised synthesis of **3.37** (Scheme 39).

One reported drawback of the above synthesis was the instability of the chloroquinazoline intermediate **3.63**. Recent improvements circumvent this issue by converting the quinazolinone **3.61** to the corresponding methylthioquinazoline **3.64** by thiolation and a subsequent methylation (route B, Scheme 39) [99]. Nucleophilic aromatic substitution with the previously used aniline derivative **3.62** then furnishes erlotinib in 73% yield. Despite adding an additional step this new procedure was found to be more robust and reproducible than the previously reported sequence (route A, Scheme 39).

**Scheme 40:**
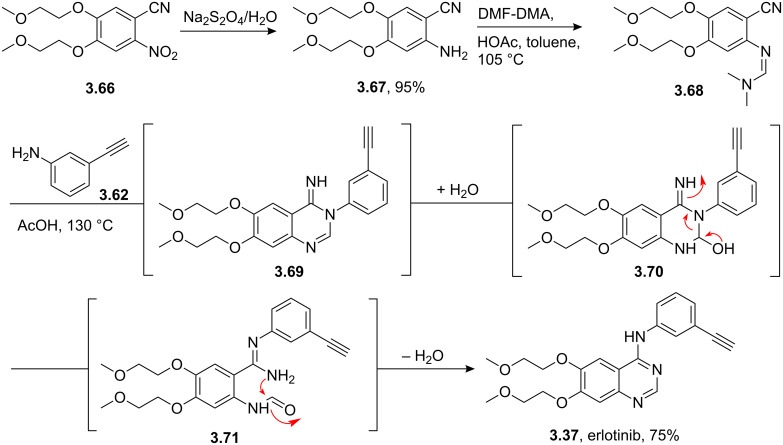
Synthesis of erlotinib proceeding via Dimroth rearrangement.

An alternative route which completely avoids the intermediacy of an activated quinazoline has also been recently reported [100] (Scheme 40). This short synthesis starts from an elaborated nitrocyanobenzene derivative **3.66** which was reduced using sodium dithionate in water to give the corresponding amine **3.67** in 95% yield. Treatment of this amine with dimethylformamide dimethylacetal (DMF-DMA) gave an *N*,*N*-dimethylformamidine **3.68** which reliably reacted with the required aniline derivative at high temperatures in the presence of a mild acid such as acetic acid. This reaction is believed to proceed via a Dimroth rearrangement, in which the intermediate iminopyrimidine **3.69** undergoes a ring-opening by addition of water. The intermediate hemi-aminal **3.70** then renders amidoaldehyde **3.71** which after cyclisation delivers **3.37**. This overall process effectively results in the perceived migration of the phenyl moiety from a pyrimidine-type nitrogen to the aniline nitrogen.

**Scheme 41:**
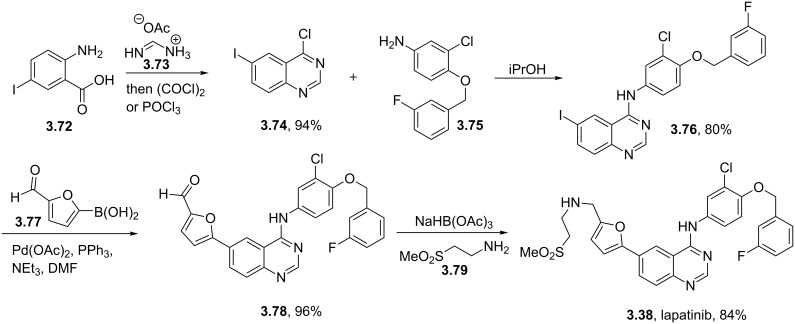
Synthesis of lapatinib.

GlaxoSmithKline’s lapatinib (**3.38**, Tykerb) is a novel dual kinase inhibitor used in the treatment of solid tumors such as those found in breast cancer and contains a quinazoline core structure. It consists of a 2,5-disubstituted furan ring, which is directly linked to the aminoquinazoline unit (Scheme 41). The quinazoline heterocycle was prepared starting from 5-iodoanthranilic acid (**3.72**) via initial condensation with formamidine acetate (**3.73**) followed by chlorination using oxalyl chloride or phosphorous oxychloride [101]. Performing a nucleophilic aromatic substitution on the chloride **3.74** with aniline **3.75** renders the extended core of lapatinib. This intermediate (**3.76**) was then coupled with 5-formyl-2-furanoboronic acid (**3.77**) using standard Suzuki cross-coupling conditions. Finally, a reductive amination of the pendant aldehyde of **3.78** with 2-(methylsulfonyl)ethylamine (**3.79**) furnishes the desired product lapatinib (Scheme 41).

**Scheme 42:**
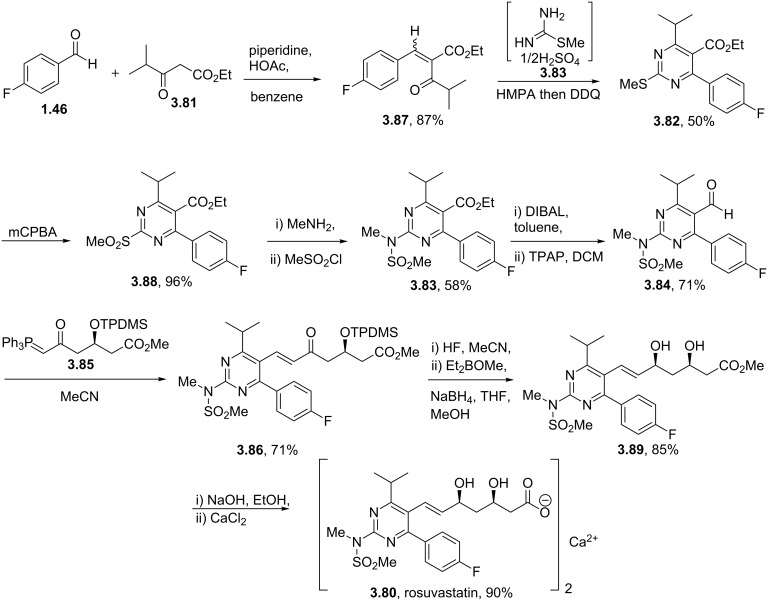
Synthesis of rosuvastatin.

Rosuvastatin (**3.80**, Crestor) is a competitive HMG-CoA reductase inhibitor that is marketed by AstraZeneca as an important cholesterol lowering drug. Like all other representatives of the statin family rosuvastatin consists of a *syn*-1,3-diol pharmacophore attached to a highly substituted heterocyclic core (Scheme 42) [102]. Various syntheses of the pyrimidine unit have been described, Hirai and Watanabe for example reported a classical cyclocondensation strategy converting 4-fluorobenzaldehyde (**1.46**) and an α-ketoester **3.81** to the desired pyrimidine system **3.82** utilising *S*-methylisothiourea hemisulfate (**3.83**) with a DDQ-mediated dehydrogenation [103–104]. Oxidation of the thioether **3.82** followed by nucleophilic aromatic substitution and subsequent mesylation installs the sulfonamide moiety **3.83**, after which the ester on the pyrimidine was converted to the required aldehyde functionality **3.84**, then allowing for the introduction of the side chain **3.85** via a Horner–Wadsworth–Emmons olefination. The newly furnished advanced intermediate **3.86** was next desilylated and subjected to a Narasaka reduction yielding rosuvastatin after saponification and formation of the calcium salt.

**Scheme 43:**
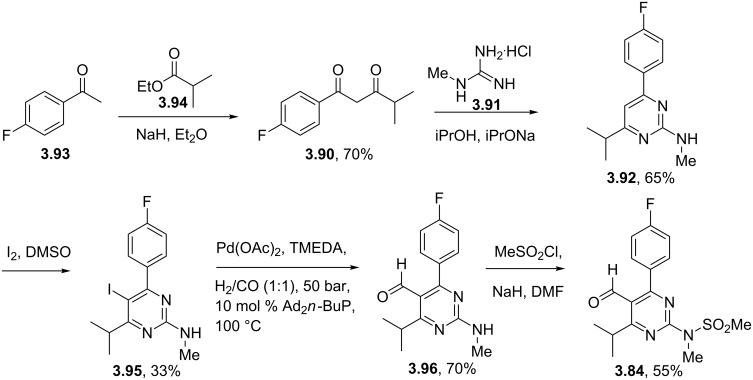
Alternative preparation of the key aldehyde towards rosuvastatin.

An alternative approach to the key aldehyde **3.84** on route to rosuvastatin has been reported which also features an *N*-methylguanidine mediated pyrimidine formation (Scheme 43) [105]. This protocol was found to work well if the condensation of the required 1,3-dicarbonyl **3.90** with the guanidine derivative **3.91** was carried out in isopropanol in the presence of sodium isopropoxide as the base. Interestingly, the use of guanidine itself also yielded the desired pyrimidine, however, subsequent methylation gave a mixture of regioisomeric products. The pyrimidine core **3.92** was activated by iodination and then subjected to a palladium mediated hydroformylation reaction. For this last step, which was based on earlier studies by the Beller group [106], it was found that careful optimisation was required with respect to the palladium source, ligand and ratio of additives. The sulfonamide formation and attachment of the *syn*-diol side chain were conducted following the previous protocol as described above. Considering the relative lengths of the two sequences and the combined yields it is difficult to make much of a case for this latter route especially considering the relative expense of the palladium and associated ligands used in the hydroformylation. In addition most of the starting materials and reagents are decidedly lower costing and easier to source when pricing the first route.

### 4. Pyrazines and piperazines

Despite pyrimidines being the dominating isoforms/isomers amongst the diazine-derived drug compounds the isomeric pyrazine and pyridazine derivatives are occasionally embodied within the top selling drugs as they can further improve physicochemical properties such as binding affinities and bioavailability [107]. Historically, the first pyrazine syntheses were reported in the late 19th century when Gutknecht observed the formation of symmetric systems by bimolecular condensation of α-aminoketones and subsequent areal oxidation [108]. Despite numerous publications since those early days it is interesting to still find elements of symmetry present in most of today’s pyrazine-derived marketed drugs.

**Figure 11:**
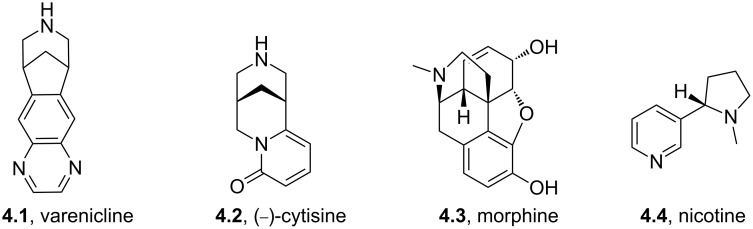
Structure comparison between nicotinic acetylcholine receptor agonists.

Varenicline (**4.1**, Chantix, Pfizer, Figure 11) is a partial agonist of certain subtypes of the nicotinic acetylcholine receptor; however, despite competitively binding to these receptors it is reported to not significantly enhance the downstream release of the neurotransmitter dopamine, thus explaining its potential in the treatment of patients addicted to smoking.

**Scheme 44:**
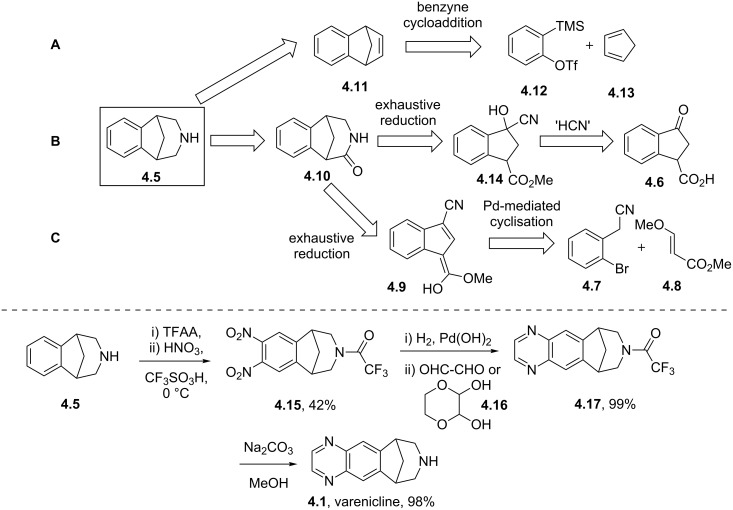
Syntheses of varenicline and its key building block **4.5**.

A comparison of the structure of varenicline with known natural nicotinic acetylcholine receptor agonists such as (−)-cytisine (**4.2**), morphine (**4.3**) or nicotine (**4.4**) shows many structural similarities, namely, a bridging/cyclic secondary or tertiary amine being placed distal to a polar aromatic substructure (Figure 11). The main difference lies in the environment of the secondary amine exemplifying commonly used tactics in designing newer and more selective inhibitors [109]. In order to prepare varenicline a common intermediate (**4.5**) has been identified and used at various stages in the history of this compound. Early routes towards this bridged tetrahydro-1*H*-benzazepine used a benzyne-cycloaddition strategy followed by OsO_4_-mediated dihydroxylation, diol-cleavage and subsequent double reductive amination (Scheme 44, pathway A). Alternatively, a homologation strategy was envisaged, where the cyanohydrin derived from carboxyindanone **4.6** was converted to the desired intermediate **4.5** via cyanide reduction, lactam formation and subsequent amide reduction (Scheme 44, pathway B). However, as both of these routes were found to be cumbersome upon scale-up due to the use of unstable and toxic reagents, a third strategy was developed utilising non-toxic commodity reagents (Scheme 44, pathway C) [110]. Therefore, 2-bromophenylacetonitrile (**4.7**) was subjected to a Michael addition with acrylate **4.8** followed by a Pd-catalysed cyclisation yielding indene **4.9** [111]. Under hydrogenation conditions this material furnished an amino-ester intermediate, which upon treatment with base produces lactam **4.10**. This material can be further reduced and protected to generate the desired building block **4.5**.

After protection of the piperidine nitrogen atom of **4.5** a regioselective double nitration using a mixture of nitric acid and triflic acid and reduction using Pearlmans catalyst is described to generate an intermediate diamine which upon immediate condensation with glyoxal and following a mild deprotection yields varenicline. Alternatively, 2,3-dihydroxy-1,4-dioxane (**4.16**) can be used instead of glyoxal under a milder set of conditions for the formation of the pyrazine ring.

**Scheme 45:**
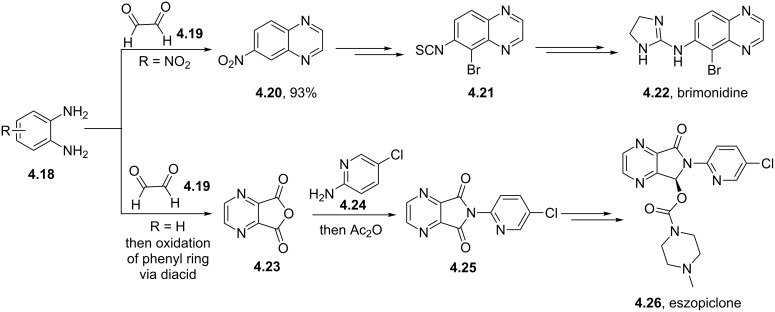
Synthetic access to eszopiclone and brimonidine via quinoxaline intermediates.

Coupling of an aromatic diamine **4.18** with glyoxal (**4.19**) to produce a quinoxaline intermediate is by far the most common approach to these molecules and is nicely illustrated in the syntheses of the antiglaucoma agent (α_2_ adrenergic agonist) brimonidine (**4.22**, Alphagan, [112] Scheme 45) and the non-benzodiazepine hypnotic eszopiclone (**4.26**, Lunesta, [113]).

**Figure 12:**
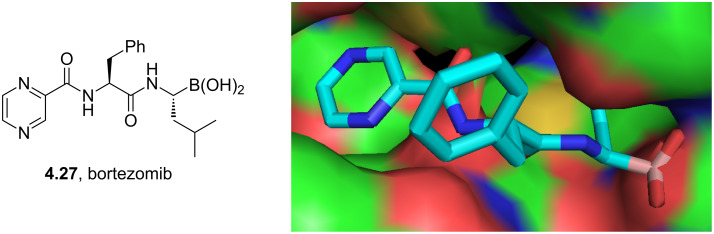
Bortezomib bound in an active site of the yeast 20S proteasome ([114], pdb 2F16).

In recent times the pyrazine-containing proteasome inhibitor bortezomib (**4.27**, Velcade) has been shown to be a promising agent for the treatment of multiple myeloma. Its structure consists of three primary building blocks: an *N*-terminal pyrazine carboxylic acid, phenylalanine and a novel boronic acid to mimic a leucine residue. Using X-ray crystallography it was established that bortezomib binds with various active sites in the yeast 20S proteasome, and displays chymotryptic, caspase-like and tryptic activities with varying degrees of binding [114]. In all of these isoforms the peptide backbone (as well as the pyrazine ring) provides multiple sites for H-bonding. The lipophilic components barely assist in binding at all but may be involved in controlling the observed selectivity towards a particular sub-type. Importantly the boronic acid binds tightly to a nearby threonine residue leading to overall excellent affinity (Figure 12).

**Scheme 46:**
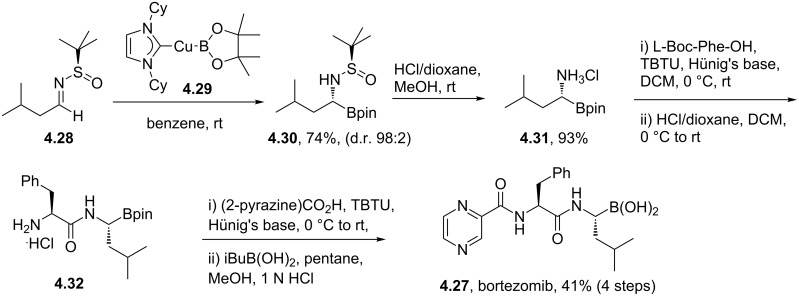
Asymmetric synthesis of bortezomib.

Since phenylalanine and the pyrazine carboxylic acid are commercially available the main synthetic challenge in the preparation of bortezomib (**4.27**) lies in the asymmetric synthesis of the boronic acid residue. Using the Ellman chemistry, *N*-*tert*-butanesulfinylaldimes (**4.28**) are readily transformed into amino boronates using a copper mediated borylation [115]. Application of this method gave the desired leucine analogue **4.31** in high yield and excellent diastereoselectivity (Scheme 46). Removal of the *N*-sulfinyl group under standard acidic conditions subsequently yields the α-amino acid **4.31** as the ammonium salt. This was subjected to standard peptide coupling protocols to furnish bortezomib in four additional steps.

**Figure 13:**
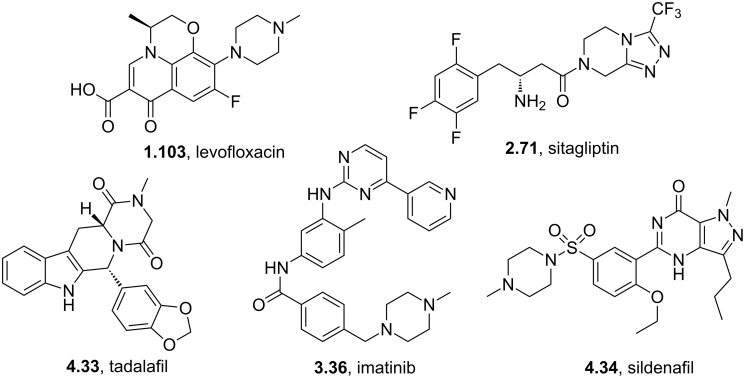
Structures of some prominent piperazine containing drugs.

Whilst the previous section would suggest pyrazines are not especially common substrates many drug substances feature the reduced piperazine ring system. For example, 2,5-diketopiperazines are typically formed by the dimerisation of amino acids. These units can be found in the well-known phosphodiesterase inhibitor tadalafil (**4.33**, Cialis, Figure 13) [7] and are prepared by this synthetic strategy. Furthermore, upon reduction of amino acid derived diketopiperazines chiral disubstituted piperazines are obtained in a concise manner. However, the majority of piperazines found in drug molecules do not contain chiral centres implying that their major purpose is to serve as linking unit enhancing binding and hydrophilicity as seen in drugs like sildenafil (**4.34**, Viagra), imatinib (**3.36**, Gleevec) or levofloxacin (**1.103**, Levaquin) [116]. Alternatively, piperazines are found in bicyclic structures like sitagliptine (**2.71**, Januvia) whose synthesis was described in the previous part of this review.

**Figure 14:**
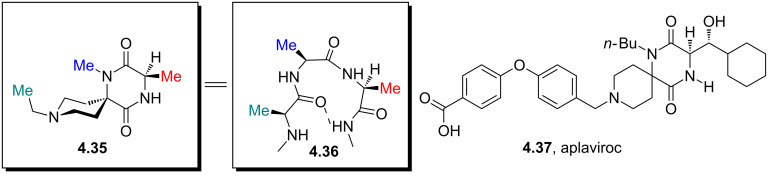
Structural comparison between the core of aplaviroc (**4.35**) and a type-1 β-turn (**4.36**).

Owing to the special properties of piperazines (increased solubility and H-bond acceptor capability etc.) it is often considered to be a privileged structure and therefore occurs widely, for instance in GlaxoSmithKlines investigational anti-HIV drug aplaviroc (**4.37**) which, despite being a promising CCR5 receptor antagonist, was discontinued due to hepatotoxicity concerns. In this compound the spirodiketopiperazine unit (**4.35**) was designed to mimic a type-1 β-turn (**4.36**) as present in G-protein coupled receptors (Figure 14) [117].

**Scheme 47:**
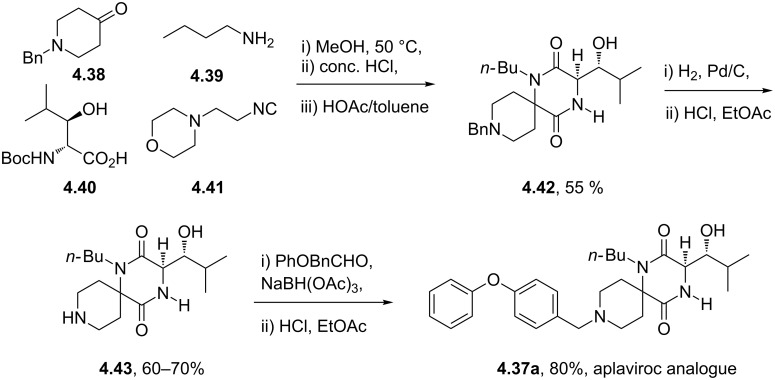
Examplary synthesis of an aplaviroc analogue via the Ugi-MCR.

The synthesis of aplaviroc and its analogues can be accomplished via the use of an Ugi multicomponent reaction (Ugi-MCR) [118]. The procedure involved the condensation of piperidone **4.38** and butylamine (**4.39**) followed by reaction of the resulting imine with isocyanide **4.41** and interception of the nitrilium intermediate with the amino acid **4.40** (Scheme 47) [119]. This sequence was completed by structural rearrangement and acid-mediated ring closure to produce the spirocyclic diketopiperazine **4.43**. Following debenzylation this material was subjected to a reductive amination finally affording aplaviroc analogues (Scheme 47).

### 5. Pyridazines and perhydropyridazines

**Scheme 48:**
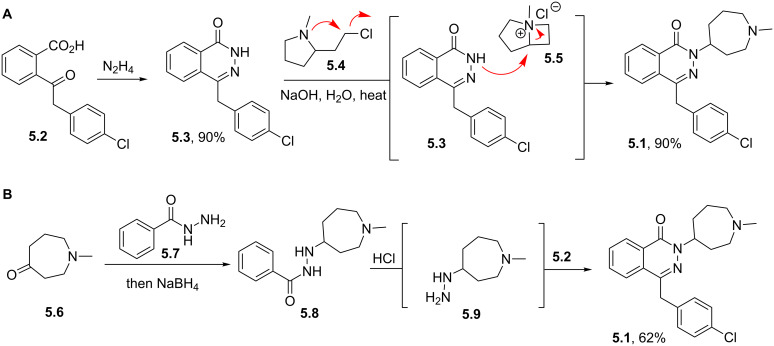
Syntheses of azelastine (**5.1**).

Structures containing pyridazine and perhydropyridazine rings are gaining interest since both nitrogen atoms can be involved with interactions with the protein target [107]. Azelastine (**5.1**) for example is a selective second generation histamine antagonist. Its novel structural feature is a saturated azepine ring attached to a phthalazinone core. A short synthesis of this molecule **5.1** has been reported which employs the coupling of hydrazine with keto-acid **5.2** to yield phthalazinone **5.3** (Scheme 48). The product when treated with 2-(2-chloroethyl)-*N*-methylpyrrolidine (**5.4**) in hot aqueous sodium hydroxide furnishes azelastine by means of an interesting ring expansion. This presumably arises through the intermediacy of [3.2.0]-scaffold **5.5** (Scheme 48, route A) [120]. Alternatively, the same keto-acid **5.2** can be subjected to a condensation with substituted hydrazine **5.9**, which was obtained from an acid-mediated hydrolysis of acyl hydrazide **5.8** (Scheme 48, route B). Although this route is longer the protocol uses a solid hydrazide **5.7** rather than volatile hydrazine as a safer alternative at scale.

**Figure 15:**
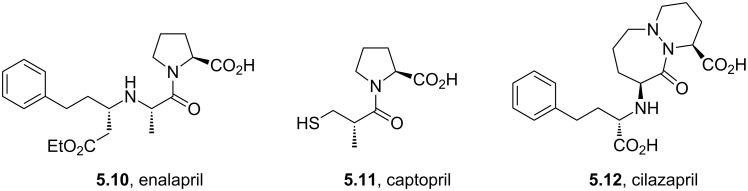
Structures of captopril, enalapril and cilazapril.

Another drug substance that contains a related heterocyclic structure is cilazapril (**5.12**), a potent inhibitor of the Angiotensine Converting Enzyme (ACE) [121]. This antihypertensive compound has a perhydropyridazine unit as part of a [5.4.0]-bicyclic system, thus apparently enhancing stability against metabolism. This unusual ring system evolved from structure optimisations of earlier ACE inhibitors enalapril (**5.10**) and captopril (**5.11**) that mimic the *N*-acylproline moiety responsible for H-bonding (Figure 15).

**Scheme 49:**
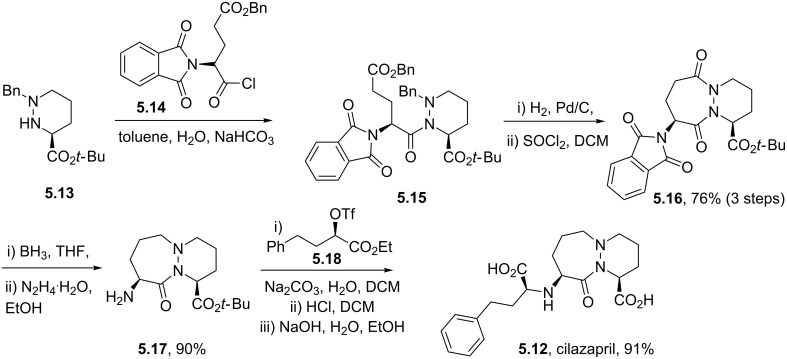
Synthesis of cilazapril.

The synthesis of cilazapril starts with a double condensation between piperazic acid derivative **5.13** and acid chloride **5.14** (Scheme 49) [122]. Upon hydrogenative cleavage of both benzyl groups and activation of the liberated carboxylic acid the bicyclic core structure **5.16** was obtained in good overall yield. A borane mediated reduction of the more accessible amide carbonyl in **5.16** followed by hydrazine induced removal of the phthalimide protecting group furnishes the primary amine **5.17**. Subsequent treatment with trilflate **5.18** yields the corresponding substitution product which through saponification of the ethyl ester and hydrolysis of the *tert*-butyl ester furnishes cilazapril (**5.12**).

### 6. Triazines and polyazacyclic systems

**Figure 16:**
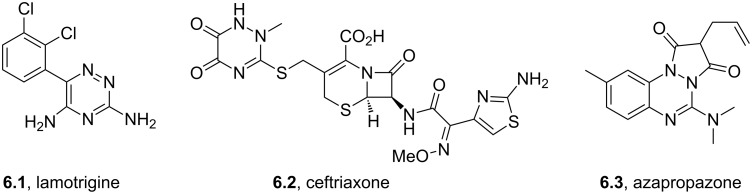
Structures of lamotrigine, ceftriaxone and azapropazone.

Triazines, both in the form of the 1,3,5-isomer and the 1,2,4-isomer, are widely represented in bioactive substances whose applications range from agrochemical to pharmaceutical usage. In general, triazines are electron-deficient compounds that readily undergo functionalisation reactions by nucleophilic aromatic substitution. They are known to participate in Diels–Alder reactions with electron-rich alkynes liberating nitrogen gas or hydrogen cyanide respectively. Lamotrigine (**6.1**, Lamictal), a sodium-channel blocker for inhibition of the release of glutamate and aspartate contains an easily recognised 1,2,4-triazine ring. Being a widely prescribed anticonvulsant, it has FDA approval for several symptoms ranging from bipolar disorder and epilepsy to general mood stabilisation. The 1,2,4-triazine unit is also present in less widely prescribed drugs such as ceftriaxone (**6.2**), a cephalosporine antibiotic and azapropazone (**6.3**), an anti-inflammatory and analgesic compound (Figure 16), again exemplifying the general scope of biological coverage.

**Scheme 50:**
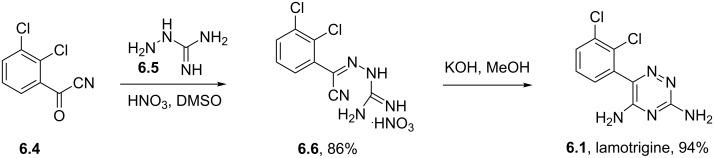
Synthesis of lamotrigine.

The reported routes to this heterocyclic scaffold are again based on condensation reactions often between an aminoguanidine and a benzoyl derivative under various acidic or basic conditions. By way of illustration 2,3-dichlorobenzoyl cyanide **6.4** reacts with aminoguanidine (**6.5**) to give an intermediate guanidinoiminoacetonitrile **6.6**, which undergoes a base-mediated cyclisation in methanol to yield lamotrigine in high overall yield (Scheme 50) [123–124].

**Scheme 51:**
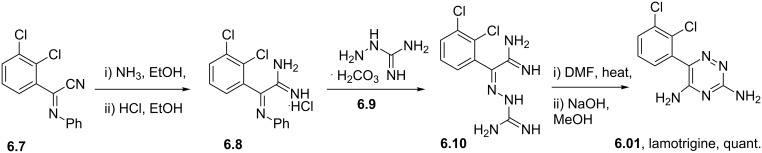
Alternative synthesis of lamotrigine (no yields reported).

Alternatively, aminoguanidine bicarbonate (**6.9**) reacts with α-(phenylimino)-2,3-dichlorophenylacetamidine hydrochloride (**6.8**) to form an α-(guanidinoimino)-2,3-dichlorophenylacetamidine salt **6.10** that can also be converted to lamotrigine under basic conditions (Scheme 51).

In the sections presented thus far the key heterocyclic moiety was either a monocyclic or benzannulated system. However, a considerable number of today’s newest drugs possess two or more annulated nitrogeneous heterocycles. Most commonly a five-membered ring such as an imidazole or pyrazole is attached to a six-membered ring containing up to four nitrogen atoms. This next section will discuss several of the most important of these compounds.

**Figure 17:**
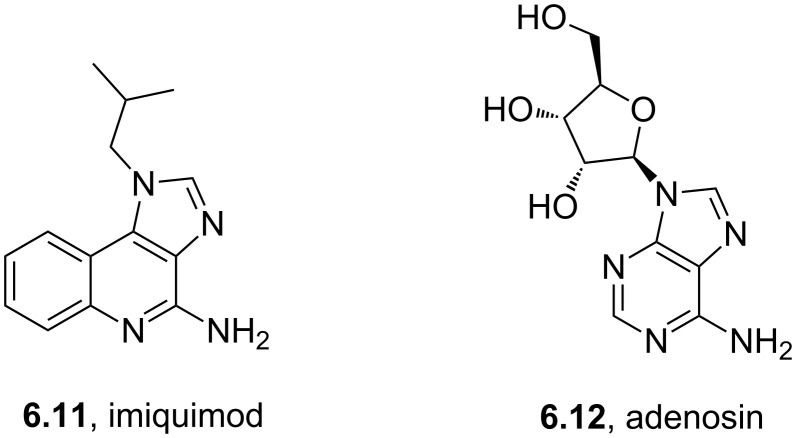
Structural comparison between imiquimod and the related adenosine nucleoside.

A first example is the antiviral compound imiquimod (**6.11**, Aldara), which consists of a fused imidazo[4,5-*c*]quinoline ring system. In this structure the isobutyl side chain mimics ribose (or deoxyribose) in the nucleoside structures such as adenosine (**6.12**, Figure 17).

**Scheme 52:**
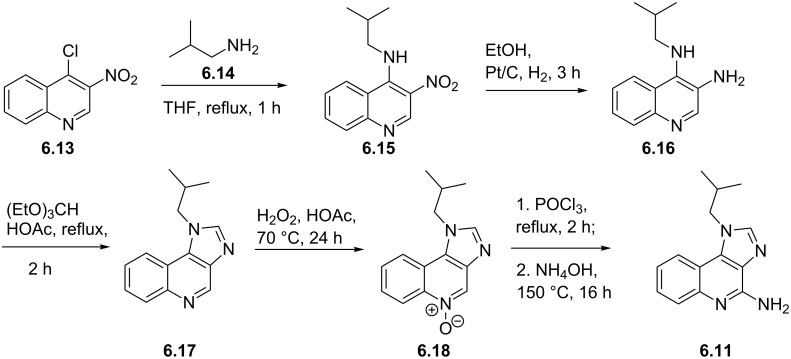
Conventional synthesis of imiquimod (no yields reported).

Imiquimod (**6.11**) can be obtained through several different routes most of which begin from a quinoline core structure followed by later installation of the imidazole ring. However, many of these early syntheses suffer from the lengthy and stepwise introduction of the various nitrogen atoms present in the core unit. An early patent route exemplifying this is depicted in Scheme 52 [125]. This sequence starts with a S_N_Ar reaction between 4-chloro-3-nitroquinoline (**6.13**) and isobutylamine (**6.14**) followed by hydrogenation of the nitro group. Upon treatment of the resulting diaminoquinoline scaffold **6.16** with triethyl orthoformate in glacial acetic acid the pendant imidazole ring is formed. In order to install the missing amino group a three step sequence involving *N*-oxide formation, chlorination and amination is required to furnish imiquimod.

**Scheme 53:**
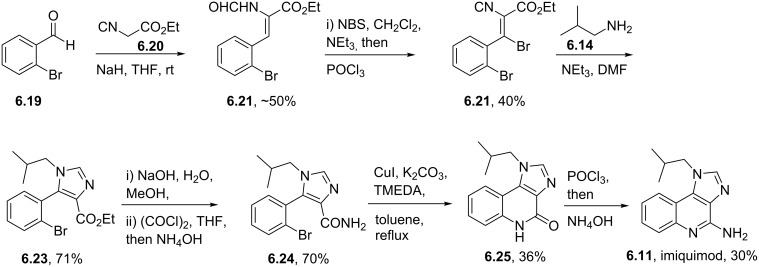
Synthesis of imiquimod.

New, more efficient routes therefore utilise different strategies. A considerably shorter and more flexible approach was described in a recent patent where 2-bromobenzaldehyde (**6.19**) was subjected to a Knoevenagel coupling reaction with ethyl isocyanoacetate (**6.20**) (Scheme 53) [126]. The resulting acrylate **6.21** was brominated under standard conditions with *N***-**bromosuccinimide and then treated with phosphorous oxychloride to reform the previously hydrolysed isonitrile moiety. Upon treatment of this material, **6.22**, with isobutylamine (**6.14**) the desired imidazole portion of imiquimod was generated. The ethyl ester in **6.23** was subsequently converted into the corresponding primary amide **6.24** as required for a copper-mediated *N*-arylation reaction rendering the imidazoquinolinone scaffold **6.25**. A phosphorous oxychloride mediated chlorination of the amide and aminolysis conclude the synthesis. Although the reported Ullmann reaction of **6.24** was low-yielding, it was not optimised using modern catalyst screening methods and could in comparison to many other related ring formations be optimised to a much higher yield [127].

**Scheme 54:**
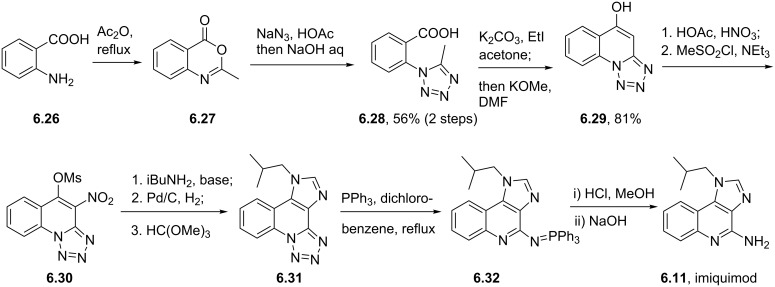
Synthesis of imiquimod via tetrazole formation (not all yields reported).

A very different approach to imiquimod (**6.11**) starts from commercially available anthranilic acid (**6.26**) which can be converted to the benzoxazine **6.27** (Scheme 54) [128]. Upon treatment of **6.27** with in situ generated hydrazoic acid the corresponding 5-methyl-1*H*-tetrazole derivative **6.28** was obtained. Esterification of the carboxylic acid followed by cyclisation under more basic reaction conditions affords the corresponding tetrazoloquinoline **6.29**. Further sequential manipulations can then be used to install the two nitrogen atoms needed for the imidazole ring. Neatly the tetrazole unit when treated with triphenylphosphine generates an intermediate iminophosphorane **6.32** via a retro-Staudinger reaction furnishing imiquimod upon work-up. While this route is synthetically very interesting it is unlikely to be amenable for transfer to bulk scale production because of the potential explosive nature of the reactive tetrazole unit.

**Figure 18:**
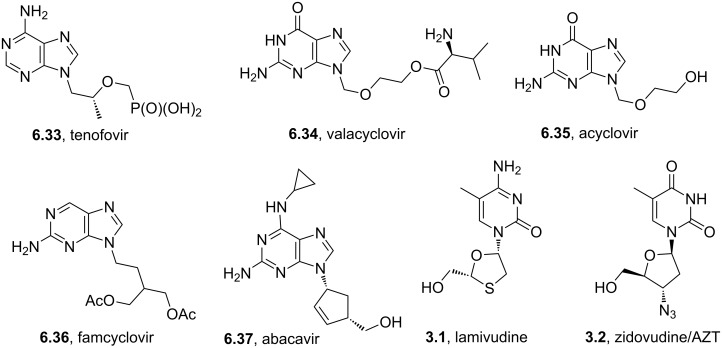
Structures of various anti HIV-medications.

Purines alongside pyrimidine structures make up the key motifs of the nucleotide bases of DNA and RNA. Currently, there are five purine containing drugs listed within the top commercial pharmaceuticals: tenofovir (**6.33**), valacyclovir (**6.34**), acyclovir (**6.35**), famcyclovir (**6.36**) and abacavir (**6.37**, Figure 18).

**Scheme 55:**
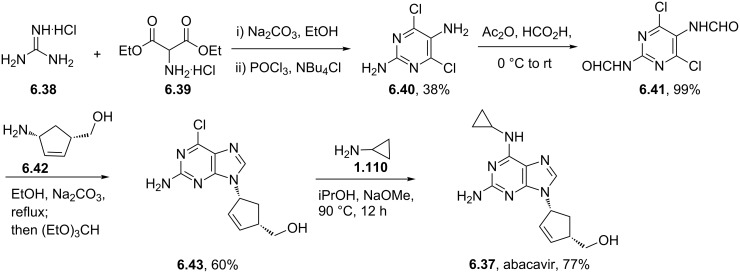
Synthesis of abacavir.

Many of these compounds are based on guanosine or adenosine as core heterocycle. They are often prescribed as combinations of individual drugs for the treatment of viral resistant strains [129]. For example, abacavir (**6.37**) exists as a cocktail component in Trizivir in combination with lamivudine (**3.1**) and zidovudine (**3.2**, AZT) or in Epzicom with lamivudine (**3.1**) only. It was found that these combinations better suppress the replication of HIV by an order of magnitude when compared to the individual drugs. The purine ring in these systems is nearly always introduced in the synthesis as a premade adenine or other purine based building block. Valacyclovir (**6.34**) is a prodrug of acyclovir (**6.35**) undergoing ester hydrolysis in vivo. Its synthesis also begins from acyclovir. For abacavir (**6.37**) the guanosine analogue comes via a stepwise process (Scheme 55) [130]. The key building block is diaminodichloropyrimidine **6.40** which can be prepared by cyclocondensation of guanidine with diethyl malonate (**6.39**), followed by chlorination using phosphorous oxychloride [131]. Formylation of both amines with formic acid in cold acetic anhydride gives rise to the *bis*-formamide **6.41**, which is then treated with aminocyclopentene **6.42** to yield the required purine ring system **6.43**. Finally, this material undergoes an S_N_Ar reaction with cyclopropylamine to furnish abacavir (**6.37**).

**Figure 19:**
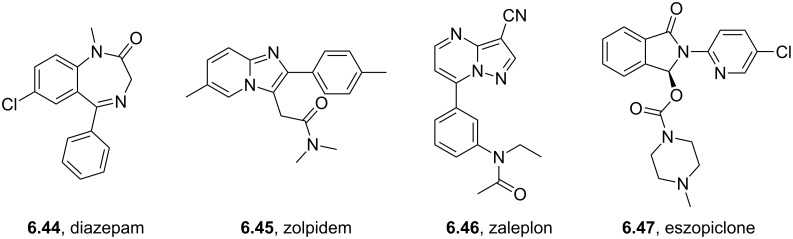
Structures of diazepam compared to modern replacements.

The benzodiazepinone ring system present in diazepam (**6.44**) is a well-recognised motif in a variety of sedatives prescribed to induce sleep in people suffering insomnia. However, more recently these structures have been superseded by species incorporating different heterocyclic scaffolds such as zolpidem (**6.45**) [6,132–134], zaleplon (**6.46**) [135] and eszopiclone (**6.47**) [7]. These structures display anxiolytic effects but induce relatively little sedation [136]. The structures of these more modern variants are obtained by scaffold hopping protocols, a widely used technique to bridge between apparently diverse compounds (Figure 19) [137].

**Scheme 56:**
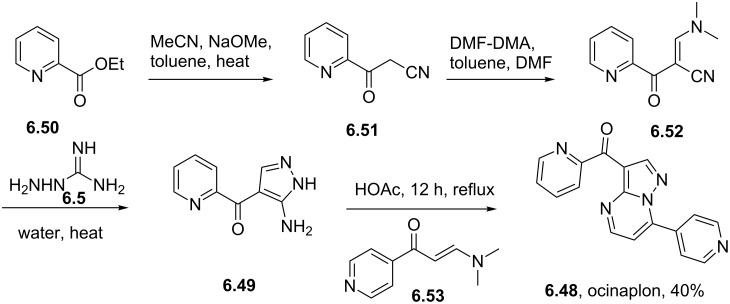
Synthesis of ocinaplon.

Amongst the novel pyrazolopyrimidine anxiolytics ocinaplon (**6.48**) has been used as an important lead compound. The patented preparation of ocinaplon [138] describes the use of vinylogous amides undergoing condensation reactions with guanidines or an aminopyrazole. In order to prepare the required aminopyrazole **6.49** an addition of acetonitrile into ethyl picolinate (**6.50**) under basic conditions followed by an aldol condensation with dimethylformamide diethylacetal (DMF-DMA) is described (Scheme 56). The resulting vinylogous amide **6.52** undergoes cyclisation with aminoguanidine (**6.5**) at elevated temperatures yielding the desired aminopyrazole **6.49**. This material is subsequently progressed to the pyrazolopyrimidine core of ocinaplon by condensation with a second vinylogous amide **6.53**, itself derived from 4-acetylpyridine and DMF.

**Scheme 57:**
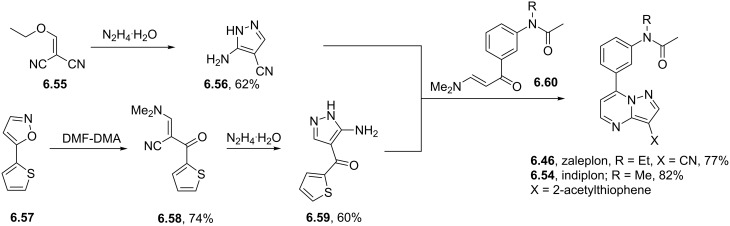
Access to zaleplon and indiplon.

Although ocinaplon has been discontinued as a drug due to complications involving the liver other members of this family have been developed using analogous strategies. Two of these alternative medications are zaleplon (**6.46**, Sonata) and indiplon (**6.54**) which jointly share the pyrazolo[1,5-*a*]pyrimidine as a central component. However, they differ in their substitution patterns (Scheme 57). The aminopyrazole in zaleplon was prepared in a straightforward manner from hydrazine and dinitrile **6.55** [139], whereas for indiplon the aminopyrazole structure comes from a ring-opening of an isoxazole, which yields the vinylogous amide **6.55**. The desired aminopyrazole **6.56** was obtained upon treatment with hydrazine in the normal fashion [140].

Typical of many drug discovery programs novelty often arises from new patentable heterocyclic scaffolds that appear to improve the compounds pharmacokinetic and pharmacodynamic properties. This was true of the pyrazolopyrimidone containing drug which was initially designed for the treatment of acute chest pain, but later turned out to be a potent PDE5 inhibitor preventing the breakdown of cGMP instead. This first-in-class compound is known as sildenafil (**6.61**, Viagra), which today is widely used for the treatment of erectile dysfunction [141].

**Scheme 58:**
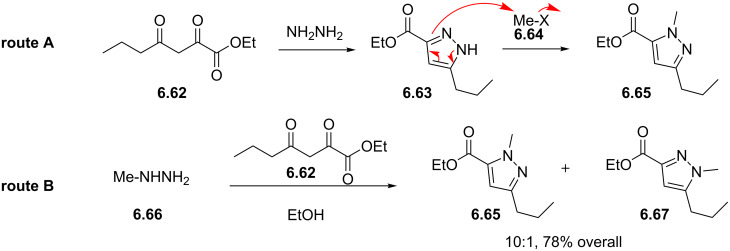
Different routes towards the required *N*-methylpyrazole **6.65** of sildenafil.

The pyrazolopyrimidone core of sildenafil imitates the purine structure of the guanidine base [142]. In most routes to the pyrazole ring a condensation reaction between a diketoester **6.62** and hydrazine begins the process. It has been found that the desired N-2 isomer **6.63** is normally the predominant tautomer in solution giving the desired N-1 alkylated pyrazole product **6.65** when performed under neutral or acidic conditions in both polar and non-polar solvents (Scheme 58, route A). Presumably, the N-2 nitrogen is donating its lone pair into the ring such that the N-1 nitrogen can react in a pyridine-like fashion with the electrophile. Under basic conditions the undesired thermodynamic N-2 methylation product **6.67** was observed [143].

Alternatively, in these reactions methylhydrazine can be used to directly generate the methylated pyrazole products (**6.65** and **6.67**; Scheme 58, route B). However, the reaction only proceeds with good regioselectivity (10:1) if the diketone **6.62** is added to a solution of methylhydrazine in ethanol. If the order of addition was reversed a 5:4 mixture of alkylation products was instead obtained.

**Scheme 59:**
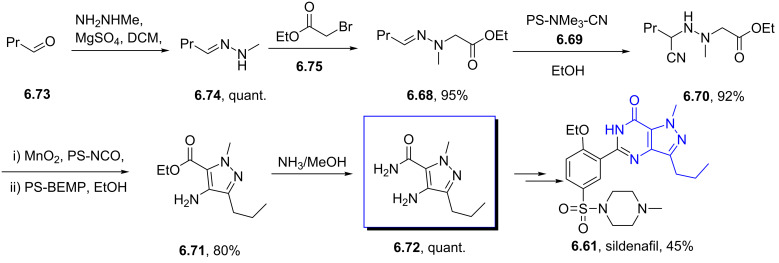
Polymer-supported reagents in the synthesis of key aminopyrazole **6.72**.

Our group has described a synthesis of sildenafil (**6.61**) in which the pyrazole core as well as the target molecule was prepared using only polymer supported reagents (Scheme 59) [144]. In this sequence a highly functionalised hydrazone **6.68** can be generated and subsequently reacted with a polymer-supported source of cyanide **6.69**. The resulting cyanohydrazine **6.70** furnishes the desired pyrazole core **6.71** on treatment with manganese dioxide under basic conditions. Continuation of this approach delivered the API in high yield and avoided laborious purification procedures at each individual step.

**Scheme 60:**
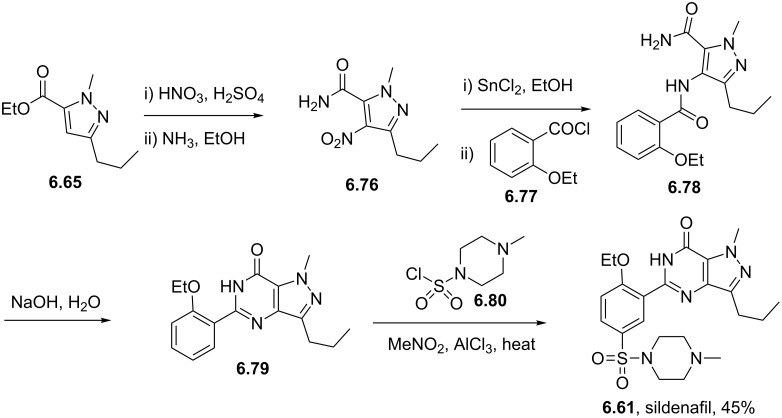
Early synthetic route to sildenafil.

In order to fulfil a process route to sildenafil (**6.61**), a nitration reaction on the premade pyrazole ring of **6.65** was performed (Scheme 60). The resulting molecule was then subjected to ammonolysis of the ester group, both of these initial two steps progressed in very high yield delivering a crystalline product. Stannous chloride in ethanol was employed to reduce the nitro group rendering an intermediate amine which was acylated with 2-ethoxybenzoyl chloride (**6.77**). Further reaction with aqueous sodium hydroxide promoted the cyclisation of the *bis*-amide **6.78** to furnish the desired pyrazolopyrimidone structure **6.79**. Finally, a Friedel–Crafts acylation of this material with sulfamoyl chloride **6.80** in the presence of AlCl_3_ was used to generate sildenafil.

**Scheme 61:**
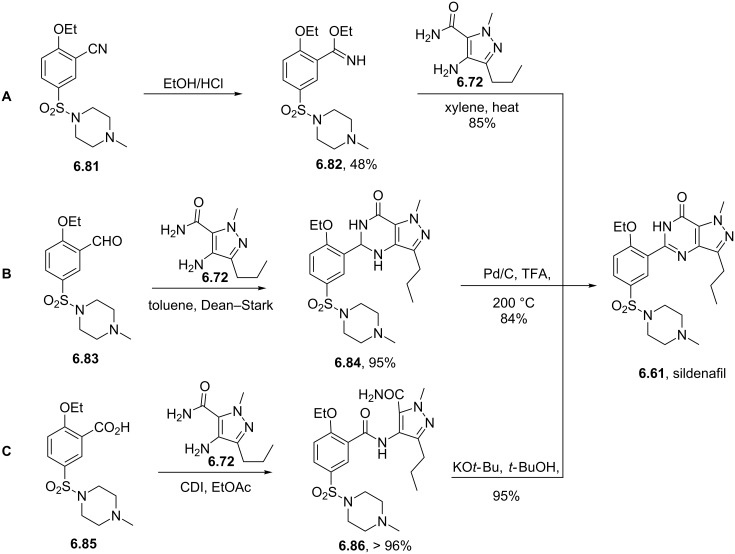
Convergent preparations of sildenafil.

More recently several new convergent routes for the preparation of sildenafil have been reported [141]. For instance, nitrile **6.81** can be converted into imidate **6.82** and subsequently cyclised to sildenafil with aminopyrazole **6.72** (Scheme 61, route A).

Alternatively, imine formation between aldehyde **6.83** and the heterocyclic amine **6.72** can be used to furnish dihydrosildenafil (**6.84**) upon intramolecular trapping of the imine intermediate. Subsequent oxidation to sildenafil can be accomplished using Pd/C and trifluoroacetic acid at elevated temperatures (Scheme 61, route B). The commercial synthesis, however, utilises a CDI-mediated amide formation between acid **6.85** and aminopyrazole **6.72**, followed by base-mediated cyclocondensation, both steps occurring with excellent yields (Scheme 61, route C).

**Figure 20:**
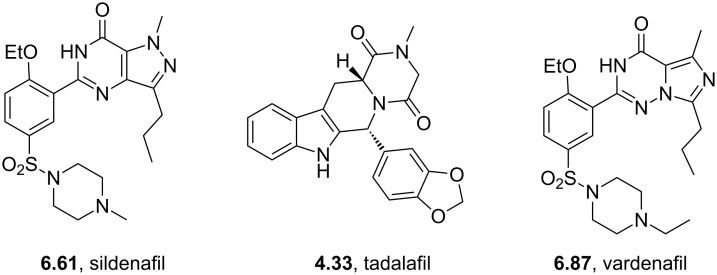
Comparison of the structures of sildenafil, tadalafil and vardenafil.

In addition to sildenafil (**6.61**) and tadalafil (**4.33**) a third phosphodiesterase inhibitor, under the name of vardenafil (**6.87**, Levitra), also features amongst the top retailing pharmaceuticals (Figure 20). The only structural difference between vardenafil and sildenafil lies in the nature of the bicyclic core mimicking the guanidine DNA base. Here vardenafil contains an imidazo[5,1-*f*][1,2,4]triazine core.

**Scheme 62:**
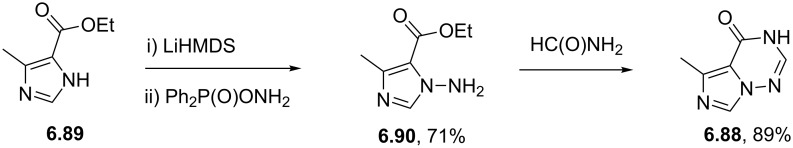
Short route to imidazotriazinones.

A recent paper describes an interesting route towards a number of imidazotriazinones **6.88** by making use of an electrophilic *N*-amination reaction of substituted 3*H*-imidazoles **6.89** followed by cyclocondensation of the intermediate **6.90** with formamide (Scheme 62) [145].

**Scheme 63:**
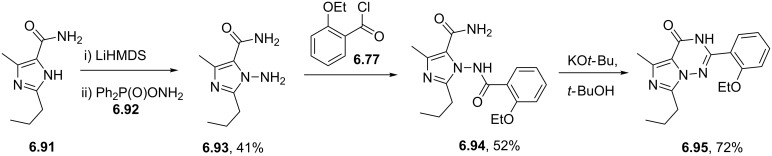
Alternative route towards vardenafils core imidazotriazinone (**6.95**).

This route was also applied to the formal synthesis of vardenafil where the amido imidazole starting material **6.91** was α-aminated using LiHMDS as base and *O*-(diphenylphosphinyl)hydroxylamine (**6.92**, Ph_2_P(O)ONH_2_, Scheme 63). The subsequent acylation with 2-ethoxybenzoyl chloride (**6.77**) provided the precursor for a base-mediated cyclocondensation to the desired imidazotriazinone **6.95** which was obtained in good yield.

**Scheme 64:**
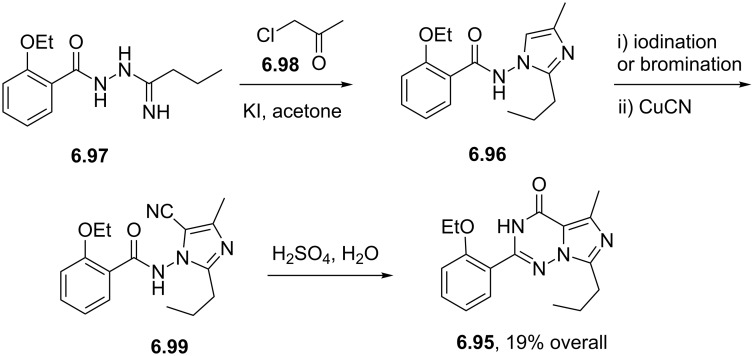
Bayer’s approach to the vardenafil core.

In an alternative approach reported by Bayer, the substituted imidazole ring **6.96** was formed via cyclisation of **6.97** in the presence of chloroacetone (**6.98**) followed by elaboration via halogenation [146] and subsequent copper-catalysed cyanation to give nitrile **6.99**. This intermediate then undergoes acid-mediated ring closure delivering the imidazotriazinone core of vardenafil (**6.95**) in a reasonable 19% yield over the 5 step sequence (Scheme 64).

**Scheme 65:**
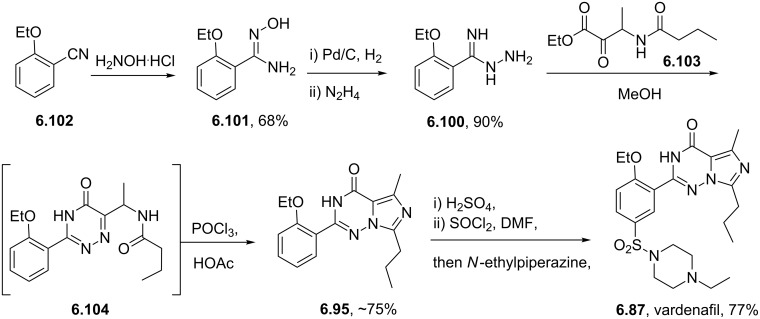
Large scale synthesis of vardenafil.

In order to generate vardenafil on a production scale the Bayer group also reported the use of a amidrazone **6.100** as a cyclisation precursor (Scheme 65). First the amide oxime **6.101** was formed from the corresponding nitrile **6.102** which upon palladium-mediated N–O bond cleavage yielded an amidine derivative on route to the desired amidrazone **6.100** through exchange with hydrazine. This material was then subjected to a condensation reaction with keto-ester **6.103** in refluxing methanol followed by a phosphorous oxychloride mediated ring closure yielding imidazotriazinone **6.95**. The synthesis of vardenafil was completed by a stepwise sulfamoylation protocol.

**Scheme 66:**
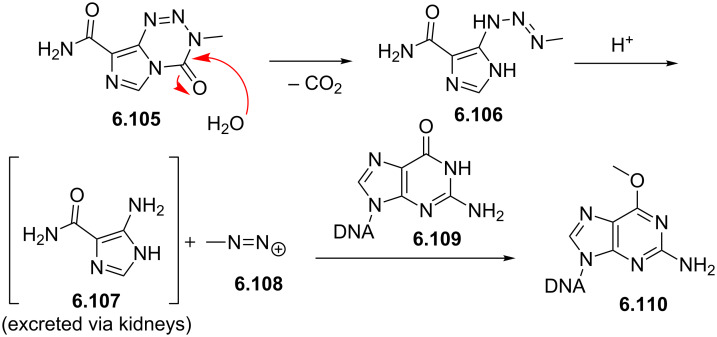
Mode of action of temozolomide (**6.105**) as methylating agent.

A structurally related drug to vardenafil is Schering’s temozolomide (**6.105**, Temodar), an antineoplastic medication consisting of an unusual imidazo[5,1-*d*]1,2,3,5-tetrazine core. Temozolomide itself is a prodrug, which after intestinal absorption can cross the blood brain barrier where it is readily hydrolysed liberating carbon dioxide and 5-(methyltriazen-1-yl)-imidazole-4-carboxamide (**6.106**), which further fragments into 5-aminoimidazole-4-carboxamide (**6.107**) and the highly reactive methyldiazonium cation (**6.108**) (Scheme 66) [147]. This methyldiazonium cation is a powerful electrophile capable of methylating guanine nucleosides at the C6 carbonyl oxygen and eventually leading to base-pair mismatch and interruption of DNA replication thus accounting for its wide use in the treatment of advanced cancers.

**Scheme 67:**
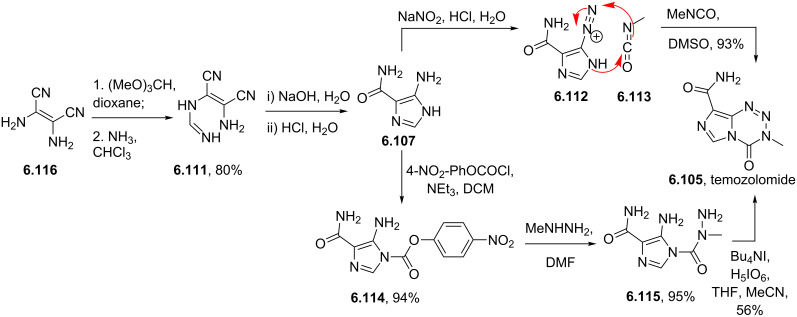
Different routes to temozolomide.

The common intermediate in the synthesis of temozolomide is 5-aminoimidazole-4-carboxamide (**6.107**) which can be prepared from *N*-(2-amino-1,2-dicyanovinyl)formamidine (**6.111,** [148–152]) under basic conditions (Scheme 67). Its diazotisation followed by reaction with methyl isocyanate (**6.113**) affords temozolomide in a non-concerted cycloaddition sequence [153]. However, due to the dangers of contamination by residual methyl isocyanate [154] this route would not be acceptable on industrial scale. Therefore in an alternative approach, 5-aminoimidazole-4-carboxamide (**6.107**) can be treated with 4-nitrophenyl chloroformate to yield carbamate **6.114** [155]. This material reacts with methylhydrazine to form semicarbazide **6.115** which can be oxidised with tetrabutylammonium iodide and periodic acid furnishing temozolomide. However, the use of highly toxic methylhydrazine also reduces the application of this route in a process setting.

**Scheme 68:**
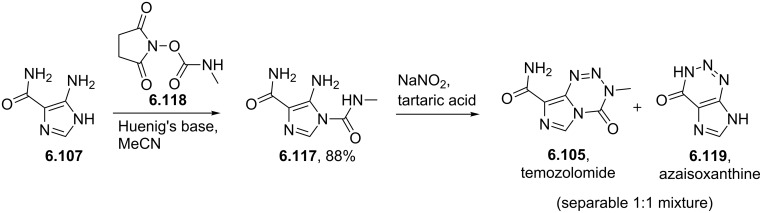
Safer route towards temozolomide.

A more safe and benign approach reports upon the high yielding conversion of key imidazole **6.107** into urea derivative **6.117** using *N*-succinimidyl-*N*’-methyl carbamate (**6.118**) (Scheme 68) [156]. Upon treatment of this material with sodium nitrite and tartaric acid temozolomide is quickly obtained, albeit as a separable 1:1 mixture with the related azaisoxanthine (**6.119**). The various reported routes towards this drug hence demonstrate the delicate balance between safety and efficiency when preparing such a compound on large scale.

When analysing the heterocyclic core structures discussed in the last part of this article, one will recognise the manifold structural differences and consequently unique modes of actions of these compounds. However, it is remarkable to see so few marketed drugs containing those novel and structurally complex heterocyclic cores. In particular the absence of heteroatoms such as oxygen and sulfur seems particularly striking considering their abundance within the previously reviewed 5- membered heterocycles.

**Figure 21:**
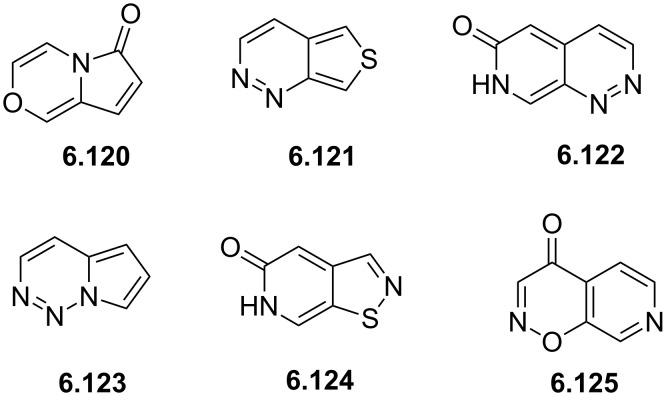
Some unreported heterocyclic scaffolds in top market drugs.

In a recent article [157] this finding was addressed and using computer based algorithms it was predicted that numerous interesting heterocycles are in fact tractable although they appear to be unknown to the synthetic community (Figure 21). With this in mind it is hoped that medicinal chemists are currently exploiting such unexplored scaffolds and that we will see them incorporated in future drug molecules.

## Conclusion

After compiling the above material a number of conclusions can be drawn regarding the abundance of certain heterocycles and the frequency and nature of typical chemical transformations applied in current drug syntheses. Amongst the aromatic heterocycles encountered in drug molecules in this review pyridines and pyrimidines are most common and are followed by purines. It is likely this results from the abundance of these heterocycles in natural products such as alkaloids and important cofactors/vitamins (pyridines) and nucleotides (pyrimidines and purines). Furthermore, this might suggest a classical approach to drug design where substrate analogues gain inspiration from existing natural ligands. The use of robust scaffold hopping methods and more advanced design parameters have inspired novel heterocylic approaches with distinct physicochemical advantages. Interestingly, many of these newer scaffolds were originally thought to be synthetically intractable and hence were overlooked for many years. Nevertheless, the drive for novel patent positions has resulted in the discovery of several new heterocyclic syntheses.

It is evident that reduced forms of parent heterocycles such as piperidines and piperazines and their beneficial features (H-bonding, bioavailability, hydrophilic spacer, pharmacophore etc.) are beginning to dominate synthesis strategies. In addition, these structures can be used easily in order to introduce asymmetry and display usable chiral information in drug molecules to improve potency.

Transformations that lead to six-membered heterocycles show high dependence on condensation reactions between carbonyl compounds and nitrogen-containing building blocks as the primary synthesis route. As such ammonia, amidines/guanidines or amidrazones become classical reaction partners with ketoesters and diketones. Due to the electron-deficient nature of many of these heterocycles nucleophilic aromatic substitutions are widely used to subsequently functionalise these ring systems. For fully reduced heterocycles more linear synthesis sequences are used in their construction often with an eye to developing asymmetric processes. Newer chemistries using metathesis or various organometallic protocols are beginning to have a more important impact. Owing to the multi-step nature which is necessary to generate novel heterocycles we should anticipate that further advances will be necessary, especially sequences that lead to ready telescoping of the routes. Without doubt the pressure currently imposed on pharmaceutical companies in order to deliver novel species more rapidly and at lower cost will drive innovation and discovery to enable many new methods.
